# Synthetic Epigenetic Reprogramming of Mesenchymal to Epithelial States Using the CRISPR/dCas9 Platform in Triple Negative Breast Cancer

**DOI:** 10.1002/advs.202301802

**Published:** 2023-05-22

**Authors:** Charlene Waryah, Joseph Cursons, Momeneh Foroutan, Christian Pflueger, Edina Wang, Ramyar Molania, Eleanor Woodward, Anabel Sorolla, Christopher Wallis, Colette Moses, Irina Glas, Leandro Magalhães, Erik W. Thompson, Liam G. Fearnley, Christine L. Chaffer, Melissa Davis, Anthony T. Papenfuss, Andrew Redfern, Ryan Lister, Manel Esteller, Pilar Blancafort

**Affiliations:** ^1^ Cancer Epigenetics Group Harry Perkins Institute of Medical Research Perth WA 6009 Australia; ^2^ Centre for Medical Research University of Western Australia Perth WA 6009 Australia; ^3^ Monash Biomedicine Discovery Institute Monash University Melbourne VIC 3800 Australia; ^4^ Department of Biochemistry and Molecular Biology Monash University Melbourne VIC 3800 Australia; ^5^ Australian Research Council Centre of Excellence in Plant Energy Biology School of Molecular Sciences University of Western Australia Perth WA 6009 Australia; ^6^ Harry Perkins Institute of Medical Research QEII Medical Centre and Centre for Medical Research, The University of Western Australia 6 Verdun St Perth WA 6009 Australia; ^7^ Population Health and Immunity Division Bioinformatics Division Walter and Eliza Hall Institute of Medical Research Melbourne VIC 3052 Australia; ^8^ Department of Biochemistry and Molecular Biology Faculty of Medicine Dentistry and Health Sciences University of Melbourne Melbourne VIC 3010 Australia; ^9^ Evolutionary Neurogenomics Swammerdam Institute for Life Sciences University of Amsterdam Amsterdam XH 1098 The Netherlands; ^10^ Postgraduate Program of Genetics and Molecular Biology Institute of Biological Sciences Federal University of Pará Belém 66075‐110 Brazil; ^11^ School of Biomedical Sciences Queensland University of Technology Brisbane QLD 4000 Australia; ^12^ Translational Research Institute Brisbane QLD 4102 Australia; ^13^ Department of Medical Biology University of Melbourne Melbourne VIC 3800 Australia; ^14^ The Kinghorn Cancer Centre Garvan Institute of Medical Research Darlinghurst NSW 2010 Australia; ^15^ St Vincent's Clinical School UNSW Medicine Darlinghurst NSW 2010 Australia; ^16^ School of Medicine University of Western Australia Perth WA 6009 Australia; ^17^ Josep Carreras Leukemia Research Institute (IJC) Badalona Barcelona 08916 Spain; ^18^ Centro de Investigacion Biomedica en Red Cancer (CIBERONC) Madrid 28029 Spain; ^19^ Institucio Catalana de Recerca i Estudis Avançats (ICREA) Barcelona Catalonia 08010 Spain; ^20^ Physiological Sciences Department School of Medicine and Health Sciences University of Barcelona (UB) Barcelona Catalonia 08007 Spain

**Keywords:** cancer epigenetics, CRISPR/dCas9 repression, epithelial‐mesenchymal transition, triple negative breast cancer, ZEB1

## Abstract

Epithelial‐mesenchymal transition (EMT) is a reversible transcriptional program invoked by cancer cells to drive cancer progression. Transcription factor ZEB1 is a master regulator of EMT, driving disease recurrence in poor‐outcome triple negative breast cancers (TNBCs). Here, this work silences *ZEB1* in TNBC models by CRISPR/dCas9‐mediated epigenetic editing, resulting in highly‐specific and nearly complete suppression of *ZEB1* in vivo, accompanied by long‐lasting tumor inhibition. Integrated “omic” changes promoted by dCas9 linked to the KRAB domain (dCas9‐KRAB) enabled the discovery of a ZEB1‐dependent‐signature of 26 genes differentially‐expressed and ‐methylated, including the reactivation and enhanced chromatin accessibility in cell adhesion loci, outlining epigenetic reprogramming toward a more epithelial state. In the *ZEB1* locus transcriptional silencing is associated with induction of locally‐spread heterochromatin, significant changes in DNA methylation at specific CpGs, gain of H3K9me3, and a near complete erasure of H3K4me3 in the *ZEB1* promoter. Epigenetic shifts induced by *ZEB1*‐silencing are enriched in a subset of human breast tumors, illuminating a clinically‐relevant hybrid‐like state. Thus, the synthetic epi‐silencing of *ZEB1* induces stable “lock‐in” epigenetic reprogramming of mesenchymal tumors associated with a distinct and stable epigenetic landscape. This work outlines epigenome‐engineering approaches for reversing EMT and customizable precision molecular oncology approaches for targeting poor outcome breast cancers.

## Graphical abstract

This study harnesses CRISPR/dCas9 epigenome‐engineering to reprogram mesenchymal‐enriched breast cancers. Key orchestrator of EMT, ZEB1 is epigenetically silenced at mRNA and protein levels. Silencing is maintained in vivo with impaired tumor growth. There is an up‐regulation of epithelial gene programs, alteration of epigenetic landscape, and a signature of 26‐differentially expressed and methylated genes predictive of prognosis in breast cancer.

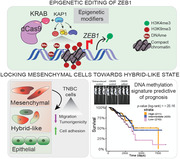

## 1 Introduction

Epithelial to mesenchymal transition (EMT) is a biological program fundamental to embryonic development and wound healing, which can be invoked by cancer cells during tumor progression.^[^ ^1^ ^]^ The EMT process, first coined by Elizabeth Hay in the 1980s in the context of embryogenesis, describes the ability of cells to turn off epithelial gene programs while acquiring mesenchymal and in some cases, stem‐like phenotypes.^[^ ^2^ ^]^ Moreover, both normal and neoplastic cells also can enter into a partial EMT cell state. Thus, cells initiating the EMT process can potentially give rise to a range of metastable intermediate or “hybrid” states along the epithelial‐mesenchymal plasticity (EMP) axis. Fundamentally, the EMT process is reversible and bi‐directional, the reverse axis being referred as mesenchymal‐to‐epithelial transition (MET).^[^ ^3^, ^4^ ^]^ In the context of epithelial cancers, the continuum array of hybrid states, with combined epithelial and mesenchymal features, confer carcinoma cells with crucial plasticity to migrate through the extracellular matrix, circulate, seed metastases, and resist therapies.^[^ ^5^, ^6^ ^]^

Cancers enriched in mesenchymal features, such as triple negative breast cancers (TNBCs) belonging to the claudin‐low and basal‐like intrinsic subtypes, are generally more proliferative, more metastatic, and often develop resistance to cytotoxic therapies.^[^ ^7^ ^]^ In contrast, cancers with more epithelial‐like features, such as luminal breast cancers, are generally more confined, less migratory, better differentiated, and express addressable therapeutic targets. Thus, the EMT process marks the transit from more benign toward more aggressive, often incurable cancers.^[^ ^8^ ^]^

The EMT program is regulated by complex signaling networks. In response to signals from changes in the tumor microenvironment, the TGF‐*β*, Notch and Wnt, signaling pathways activate a key core of pro‐mesenchymal transcription factors (EMT‐TFs), which ultimately drive EMT by transcriptional and epigenetic mechanisms. The EMT‐TFs ZEB1, ZEB2, SNAIL (SNAI1), SLUG (SNAI2), TWIST1, and TWIST2 bind E‐box sites in the target gene promoters and recruit downstream epigenetic modifiers to silence pro‐epithelial adhesion molecules, including E‐cadherin (CDH1), a critical adhesion and structural maintenance protein.^[^ ^9^, ^10^, ^11^, ^12^ ^]^ The EMT‐TFs also induce the upregulation of multiple mesenchymal markers including matrix degrading metalloproteinases, and the cell adhesion proteins N‐cadherin (CDH2) and cytoskeletal protein Vimentin (VIM).^[^ ^10^, ^13^ ^]^

Despite intense research, the non‐redundant roles of these core TFs in inducing epithelial to mesenchymal shifting of carcinoma cells remains poorly understood. The TF ZEB1 (Zinc finger E‐box‐binding homeobox 1) is a transcriptional repressor that is fundamental for the successful completion of EMT, thus driving metastatic dissemination of breast cancers.^[^ ^14^ ^]^ ZEB1 is frequently overexpressed in mesenchymal cancers and promotes metastasis, chemoresistance^[^ ^15^ ^]^ and cancer stem cell‐like behavior.^[^ ^16^ ^]^ The ZEB1 TF represses the pro‐epithelial miR‐200 microRNA family, which in turn target the *ZEB1* transcript, generating a negative feedback loop that is important for the establishment of cell fate.^[^ ^14^ ^]^ Furthermore, in many mesenchymal cancers, pro‐epithelial miRNAs are silenced through DNA and histone modifications while pro‐mesenchymal TFs are upregulated.^[^ ^17^, ^18^ ^]^

The epigenetic nature of the processes underpinning EMT provides an opportunity to restore control over the regulatory networks, reverting EMT. This introduces the possibility that highly aggressive cancers may be switched toward more benign counterparts. Furthermore, because some epigenetic modifications, such as DNA methylation (DNAme), are maintained through cell generations, epigenetic approaches to treat cancer have the potential to be long‐lasting.

The Clustered Regularly Interspaced Short Palindromic Repeats (CRISPR) and CRISPR‐associated protein 9 (Cas9) adapted for epigenome editing purposes represents a currently unexplored precision medicine tool potentially capable of reverting the EMT process. Conventional genome editing via the CRISPR/Cas9 system is catalyzed by the Cas9 endonuclease recruited in the genome by a chimeric single guide RNA (sgRNA) which is complementary to the target DNA sequence flanking a protospacer adjacent motif (PAM); the resulting complex subsequently induces a double‐stranded break (DSB).^[^ ^19^, ^20^, ^21^, ^22^, ^23^ ^]^ For epigenome editing, mutations in the nuclease domains of Cas9 produce a catalytically inactive “dead” Cas9 or dCas9. The dCas9 protein functions as a DNA‐binding protein guided by sgRNA to target the genomic sequence but it purely acts as a scaffold to ferry or recruit transcriptional effector domains to manipulate gene expression.^[^ ^21^, ^24^, ^25^, ^26^, ^27^, ^28^ ^]^

Herein, we investigate for the first time the capacity of the CRISPR/dCas9 system fused to a potent transcriptional repressor, the Krüppel associated box (KRAB) domain, to modulate the phenotypic plasticity of TNBCs belonging to the claudin‐low subtype which are enriched in mesenchymal characteristics, cancer stem cell features and are often resistant to chemotherapy. When fused to different scaffolds, such as dCas9, Zinc Fingers or Transactivator Like Effectors, the KRAB domain recruits epigenetic modifiers, such as histone methyltransferases and histone deacetylases, thus facilitating the editing of the epigenetic state at the target promoters.^[^ ^29^ ^]^ Here we demonstrate that CRISPR/dCas9‐KRAB mediated repression of *ZEB1* is sufficient to shift mesenchymal to epithelial features and to induce widespread epigenetic reprogramming, inducing long‐lasting changes in the transcriptome, and in the chromatin structure of TNBC cells. Importantly, the dCas9‐KRAB construct also induced significant changes in DNAme, including hypermethylation at CpG sites in the *ZEB1* promoter, accompanied by demethylation in several epithelial gene promoters.

This work demonstrates that targeted epigenome engineering via dCas9‐KRAB is sufficient to induce a near complete silencing of currently “hard to drug” TFs, such as ZEB1. This silencing reprograms the epigenetic landscape of mesenchymal cells, reversing EMT programs, and generating a distinct “hybrid‐like” epigenetic state that is also present in human breast tumors. Furthermore, this epigenetic reprogramming also induced changes in cell migration and reduced tumorigenesis in vivo. Genomic analyses of CRISPR‐edited cells revealed a signature of differentially expressed and methylated genes upon *ZEB1* silencing associated with gain of epithelial features. Cross‐examination with the breast cancer databases demonstrates that this epigenetic signature is also predictive of prognosis in breast cancer. This work outlines CRISPR/dCas9 systems as novel precision medicine agents able to reverse EMT by epigenetic reprogramming of TNBCs, and potentially many other malignancies associated with EMT, such as bladder and lung cancers.

## 2 Results

### 2.1 Guide‐Dependent ZEB1 Silencing of dCas9‐KRAB in Mesenchymal Breast Carcinoma Cells

**Figure 1:**
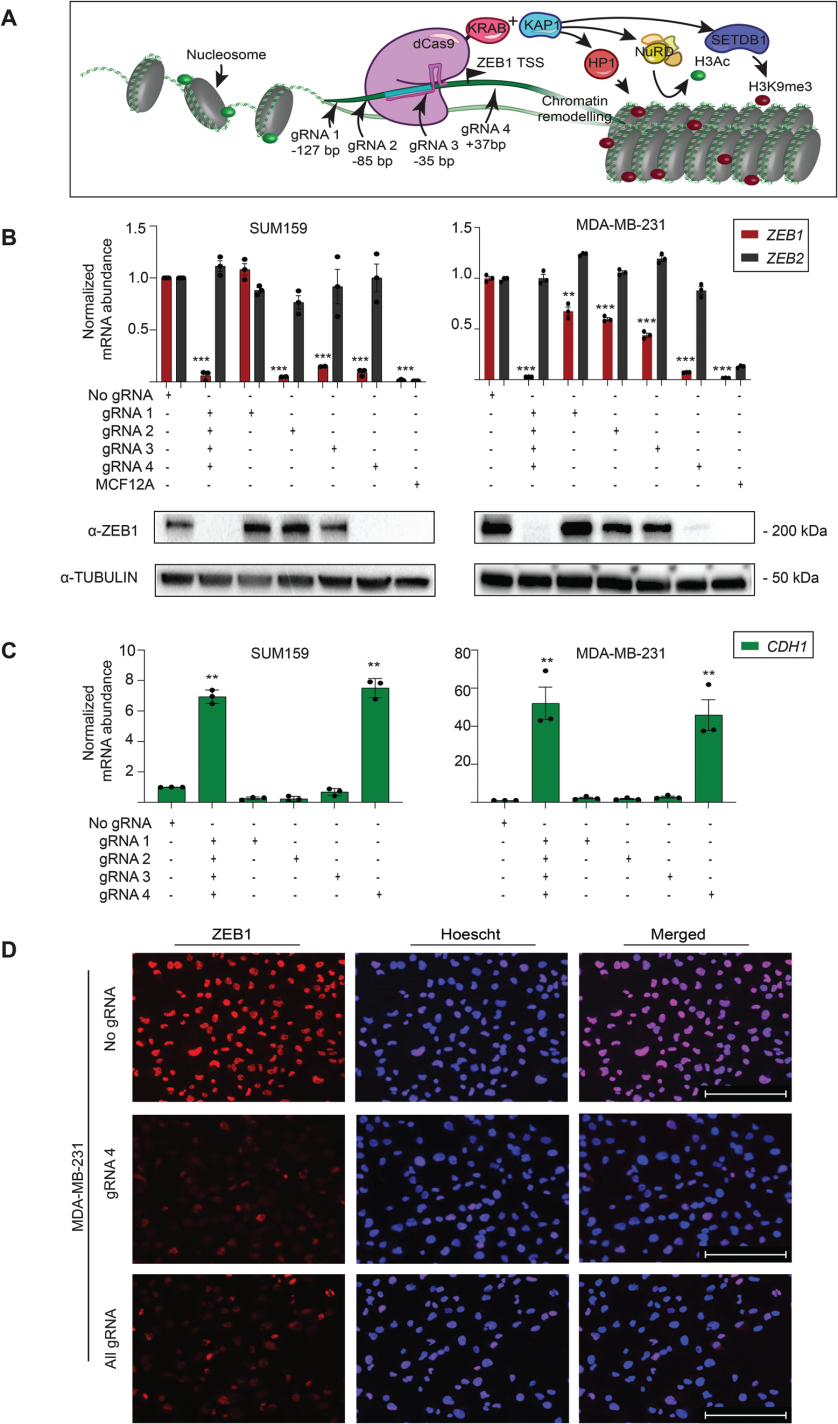
CRISPR‐dCas9‐KRAB systems targeted to the *ZEB1* promoter leads to silencing at both mRNA and protein levels. (A) Schematic representation of dCas9 fused to the Krüppel Associated Box (KRAB) domain targeted to *ZEB1* proximal promoter by four gRNAs. Locations of the gRNA (in base pairs, bps) are depicted upstream or downstream (+/−) of the transcriptional start site (TSS). KRAB recruits the corepressor KAP1 which mediates downstream recruitment of Heterochromatin Protein alpha (HP1*α*, mediating chromatin remodeling), SET Domain Bifurcated Histone Lysine Methyltransferase 1 (SETDB1, histone methylation) and nucleosome remodeling and deacetylase complex (NuRD complex, histone deacetylation). (B) Silencing of *ZEB1* mRNA expression assessed by qRT‐PCR and ZEB1 protein expression by western blotting. Relative mRNA expression was quantified to No gRNA control in SUM159 and MDA‐MB‐231 cells, ***p ≤ 0001. The breast line MCF12A is included as a normal control cell line for expression of epithelial markers. Error bars represent S.E.M. (C) Upregulation of *CDH1* mRNA expression assessed by qRT‐PCR relative to No gRNA control in SUM159 and MDA‐MB‐231 cell lines, ***p ≤ 0.001. Error bars represent S.E.M. (D) Immunofluorescence for the intracellular visualization of ZEB1 expression (*red*) in gRNA 4 and All gRNA dCas9‐KRAB transduced MDA‐MB‐231 cells; Hoechst stain (*blue*) is indicated to label the nuclei. TSS, transcription start site; bp: base pair; gRNA, guide RNA; KRAB: Krüppel associated box; KAP1, Krüppel associated protein; *CDH1*, E‐cadherin.

To specifically repress *ZEB1* gene transcription, we took advantage of catalytically‐inactive Cas9 (dCas9) C‐terminally fused to a KRAB domain, which has demonstrated potent repression of targeted genes associated with negligible off‐target activity.^[^ ^26^, ^29^ ^]^ When directed to the targeted proximal promoter via 20‐nucleotide guide RNAs (gRNAs), KRAB associates with its cofactor KAP1 to mediate the recruitment of HP1, SETDB1 and the NuRD complex, catalyzing chromatin remodeling, H3K9me3, and removal of H3 acetylation, respectively, resulting in robust suppression of gene expression^[^ ^56^, ^57^, ^58^ ^]^ (**Figure** **1**A). The capacity of CRISPR to induce *ZEB1* repression was assessed in two human TNBC cell lines, SUM159 and MDA‐MB‐231, representing models for the claudin‐low mesenchymal subtype of breast cancer.^[^ ^59^ ^]^

Four gRNAs^[^ ^1^, ^2^, ^3^, ^4^ ^]^ were designed to target the *ZEB1* proximal promoter with high predicted specificity and efficiency scores, assessed by available tools^[^ ^31^ ^]^ (Table S1A, Supporting Information). Selected gRNAs were co‐expressed with dCas9‐KRAB by a single lentiviral vector pLV dCas9‐KRAB which was transduced into the MDA‐MB‐231 and SUM159 TNBC lines either as individual gRNAs or as combination of all gRNA (All gRNA).^[^ ^25^ ^]^ Following puromycin selection, ZEB1 silencing was examined by qRT‐PCR and immunoblotting, with data normalized to the dCas9‐KRAB vector in the absence of targeting gRNA/s (dCas9‐KRAB No gRNA) (Figure 1B). The “normal‐like” (non‐transformed) MCF12A epithelial cell line expressing negligible *ZEB1* levels was included as an epithelial control reference. The qRT‐PCR data revealed that the All gRNA pool (*p*‐value < 0.001) and gRNAs 2, 3, and 4 were able to repress mRNA *ZEB1* expression in SUM159 cells relative to No gRNA with efficiencies of 0.09‐, 0.02‐, 0.10‐, and 0.11‐fold, respectively (*p*‐value < 0.05). Furthermore, the near complete silencing *ZEB1* observed with dCas9‐KRAB and gRNA 4 in SUM159 cells was significantly superior than that of dCas9 in absence of effector domain (dCas9 No eff) and gRNA 4 (0.93‐fold vs 0.73‐fold repression, respectively, *p‐*value < 0.02), indicating that dCas9 was not acting only by (passive) transcriptional interference, and that more robust silencing of *ZEB1* required the presence of the KRAB domain (Figure S1, Supporting Information).

Similarly, in MDA‐MB‐231 cells, the All gRNA pool and gRNAs 1–4 repressed *ZEB1* gene expression (0.03‐fold, *p*‐value < 0.0001, and 0.67‐, 0.60‐, 0.44‐, and 0.07‐fold, respectively, *p*‐value < 0.02). The same gRNAs had no significant effect on regulating *ZEB2* mRNA levels in the same cell lines (Figure 1B). To more comprehensively assess the specificity of the gRNAs tested with dCas9‐KRAB, we further bioinformatically identified the top predicted off‐targets using the Azimuth and Elevation algorithms^[^ ^30^, ^44^ ^]^ and experimentally investigated the expression of any nearby genes. There were no significant changes in transcript abundance (Figure S2A,B, Supporting Information) as assessed by RNA‐sequencing (RNA‐seq) suggesting negligible transcriptional modulation at predicted off‐target genes. Interestingly, immunoblotting experiments in both cell lines demonstrated that only All gRNA pool and gRNA 4 strongly suppressed ZEB1 at a protein level (Figure 1B). Thus, while gRNAs 1 (in MDA‐MB‐231 only), 2, and 3 showed significant downregulation at mRNA level, these individual gRNAs did not silence ZEB1 at protein level. There are several molecular mechanisms such as relative rates of transcription and translation, half‐lives and/or stability of mRNA and protein, that could explain the lack of correlation in mRNA and protein levels,^[^ ^60^ ^]^ however this phenomena could also reflect influence of these gRNAs with post‐transcriptional mechanisms, and this is currently under investigation in our laboratories.

The downregulation of *ZEB1* in MDA‐MB‐231 cells by dCas9‐KRAB All gRNA and gRNA 4 was sufficient to induce expression of pro‐epithelial target E‐Cadherin (*CDH1*), increasing transcript abundance by 51.98‐ and 45.84‐fold, respectively (*p*‐values < 0.005). Within the SUM159 line, dCas9‐KRAB effects with the All gRNA pool and gRNA 4 induced expression of *CDH1* to a lesser extent, driving 6.94‐ and 7.51‐fold increases in transcript abundance, respectively (*p*‐values < 0.005; Figure 1C). Immunofluorescence assays in the same stable cell lines further confirmed a very strong nuclear ZEB1 protein downregulation in MDA‐MB‐231 (Figure 1D) and SUM159 (Figure S3A, Supporting Information), respectively.

### 2.2 ZEB1 Repression Induces Epithelial‐Like Phenotypic Features

**Figure 2:**
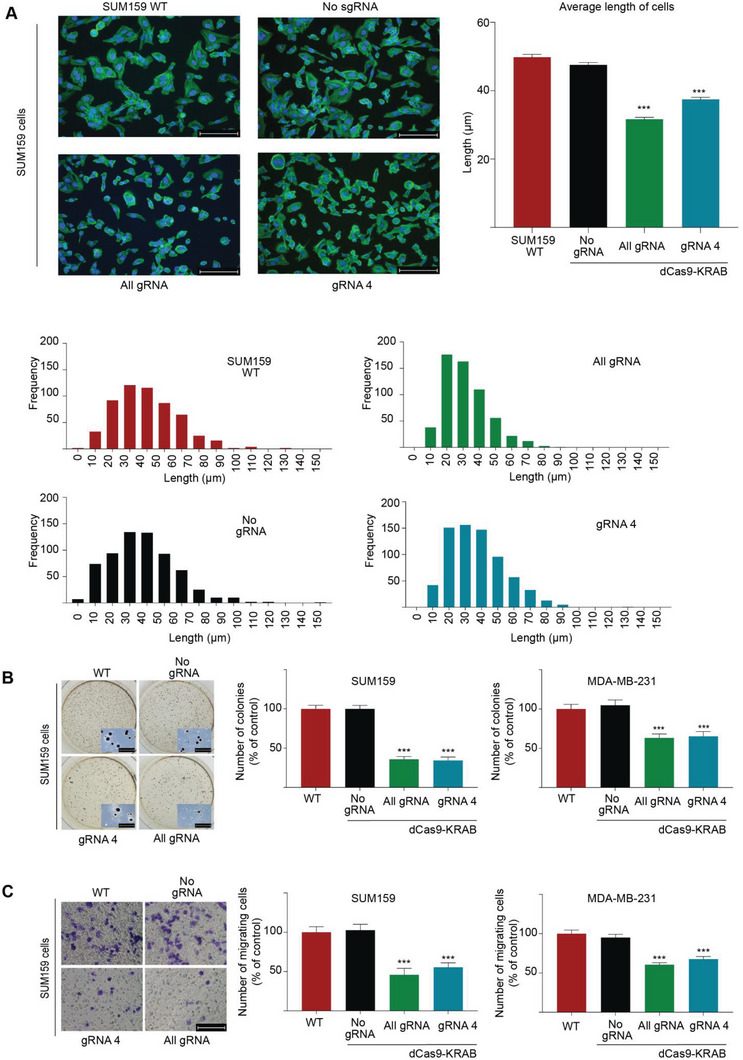
CRISPR‐dCas9 silencing of *ZEB1* reprograms the mesenchymal phenotype, inducing cell morphology changes, reduced migration and impaired colony formation. (A) Phalloidin immunofluorescence for the visualization of F‐actin in SUM159 wild type cells (untransduced), cells transduced with dCas9‐KRAB with no gRNA (No gRNA), or in presence of gRNA 4, or with All gRNAs. Average length of cells for each transduced population (*n = 565*) was measured on the major length axis, ***p ≤ 0.001. Error bars represent S.E.M. (B) Inhibition of anchorage‐independent cell growth by soft agar colony formation assays (representative images and detail of the colonies is included). Number of colonies per well are plotted as % of control for gRNA 4 and All gRNA in SUM159 and MDA‐MB‐231, respectively. Data is normalized to untransduced wild type and presented as mean values where error bars represent S.E.M, ***p ≤ 0.001. (C) Inhibition of cell invasion by Boyden migration chambers in the same cell lines. Representative images for SUM159 are displayed, along with quantification of the number of migrating cells for SUM159 and MDA‐MB‐231 cells relative to untransduced wild type cells. Error bars represent S.E.M, ***p ≤ 0.001. KRAB: Krüppel associated box. WT: wild type untransduced group.

We next investigated the phenotypic consequences of *ZEB1* silencing by the CRISPR/dCas9 systems in mesenchymal breast cancer cells. Despite the more moderate *CDH1* transcript abundance changes in the SUM159 cells, following lentiviral delivery, transduced cells showed a remarkable loss of mesenchymal features and conversely, a gain of more epithelial‐like characteristics, as assessed by immunofluorescence imaging with Phalloidin stain to assess F‐actin expression (**Figure** **2**A). While SUM159 cells exhibited a characteristic spindle‐like mesenchymal cell morphology with an average major axis length of 49.8 ± 0.78 µm (untransduced SUM159 wild type cells) and 46.74 ± 0.83 µm (dCas9‐KRAB No gRNA), the dCas9‐KRAB All gRNA and gRNA 4 transduced cells significantly transitioned to a more circular epithelial‐like shape with average diameters of 31.75 ± 0.57 µm for All gRNA and 38.26 ± 0.73 µm for gRNA 4 (*p*‐values < 0.001) (Figure 2A). Representative images of MDA‐MB‐231 are shown in Figure S3B, Supporting Information.

We subsequently investigated whether targeted *ZEB1* repression was associated with a functional reprogramming of the cancer cell phenotype. Both dCas9‐KRAB All gRNA and gRNA 4 transduced cells significantly reduced anchorage independence as assessed by soft agar colony formation assays by 65.6% and 64% respectively, relative to No gRNA (*p*‐values < 0.0001) in MDA‐MB‐231 cells, and by 34.7% (*p*‐values < 0.0001) and 36.8% (*p*‐values < 0.001) respectively, in SUM159 cells (Figure 2B). Similarly, both dCas9‐KRAB All gRNA and gRNA 4 significantly reduced cell migration relative to the dCas9‐KRAB No gRNA by 45.9% and 55% (*p‐*values < 0.001) respectively, in SUM159 wild type cells and by 60.5% and 67.7% (*p*‐values < 0.0001) respectively, in MDA‐MB‐231 wild type cells (Figure 2C).

To gain insight into single cell heterogenicity of mesenchymal versus epithelial phenotypes upon the silencing of ZEB1, we first assessed the expression of Cytokeratin 19 (CK19, epithelial marker) and Vimentin (VIM, mesenchymal marker) by immunofluorescence (IF) in MDA‐MB‐231 and SUM159 cells by comparing All gRNA, No gRNA and untransduced conditions (Figure S4A–D, Supporting Information). MCF7 cells were stained as positive and negative controls for CK19 and VIM, respectively (Figure S4B, Supporting Information). In the MDA‐MB‐231 cells there was a slight decrease in the % of VIM+ cells from 97.9 ± 2.9% (untransduced) and 98.6 ± 1.3% (No gRNA) versus 88.3 ± 6.3% (All gRNA), *p = n.s* (Figure S4A+C), Supporting Information. Conversely, as expected from inducing epithelial features, the % of CK19+ cells increased from 7.1 ± 0.8% (untransduced) and 5.5 ± 6.2% (No gRNA) up to 28.3 ± 1.6% (All gRNA), *p <* 0.008 and *p <* 0.01, respectively (Figure S4A+C, Supporting Information). Interestingly, only a relatively small proportion of hybrid cells stained positive for both markers in the All gRNA condition (≈22% of the edited cells). In SUM159 cells while there was no increase in CK19, there was a reduction in the staining intensity of VIM+ cells in the All gRNA condition relative to controls between WT versus All gRNA, *p =* 0.01 and No gRNA versus All gRNA, *p =* 0.017, respectively (Figure S4D, Supporting Information, staining intensity *at right*). Last, the expression of E‐cad was not detectable at protein level in both cell lines by IF methods upon ZEB1 silencing (Figure S6, Supporting Information), despite up‐regulation observed at mRNA level, suggesting that the re‐expression of the E‐cad promoter was not sufficient to yield detectable protein expression (Figure 1C).

### 2.3 Repression of ZEB1 Suppresses Tumor Growth In Vivo

**Figure 3:**
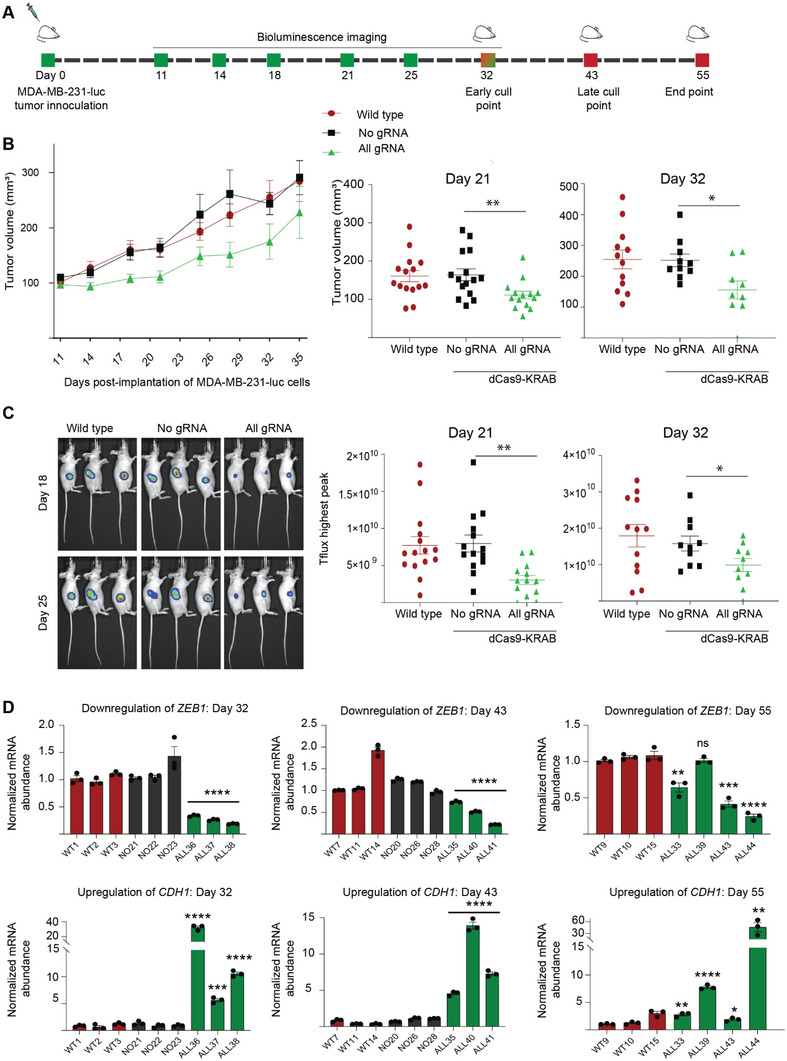
Tumor‐intrinsic repression of ZEB1 in vivo suppresses the growth of breast cancer xenograft tumor models. (A) Schematic representation of the experimental time‐line. MDA‐MB‐231 cells labeled with a luciferase gene (MDA‐MB‐231‐luc) were transduced with either dCas9‐KRAB in absence of gRNA (No gRNA) or with dCas9‐KRAB with all designed gRNAs (All gRNA). Wild type refers to untranduced cells (MDA‐MB‐231‐luc with no dCas9‐KRAB transduction). 2 × 10^6^ cells were implanted into the flank of BALB/c nude mice (*n = 15* mice per group) and tumor growth monitored by caliper measurement and bioluminescence imaging (BLI). Mice (*n = 3*) were euthanized at day 32 for an “early” and at day 43 for a “late” time‐point for histological assessment; day 55 refers to experimental end‐point (volume of the tumors >1000 mm^3^). Bioluminescence was quantified at days 11, 14, 18, 21, 25, and 32 post‐implantation of the cells. (B) Tumor growth inhibition in vivo for the MDA‐MB‐231‐luc xenograft model assessed by caliper measurements, starting at day 11 post‐implantation when the tumor growth is ≈100 mm^3^ (*at left*). Scatter dot plots outlining the decrease in tumor volume at day 21 and day 32 post‐implantation of cells are indicated (*at right*) *p ≤ 0.05, ** *p* ≤ 0.01. Error bars represent S.E.M. (C) Representative bioluminescence images of mice in un‐transduced wild type and No gRNA versus All gRNA group captured at days 18 and 25. Tflux peaks are plotted at day 21 and day 32 post inoculation, * *p* ≤ 0.05, *** *p* ≤ 0.001. Error bars represent S.E.M. (D) Expression of *ZEB1* and *CDH1* assessed by qRT‐PCR from RNA extracted from tumors, each individual tumor is indicated separately, *p ≤ 0.05, **p ≤ 0.01, ***p ≤ 0.001, ***p ≤ 0.0001, ns = non‐significant. Error bars represent S.E.M. WT: untransduced wild type group; NO: No gRNA; ALL: All gRNA; KRAB: Krüppel associated box.

To assess tumor growth dynamics in a mouse model of breast cancer, MDA‐MB‐231 cells were lentivirally engineered with a luciferase reporter, enabling non‐invasive monitoring of tumor growth by bioluminescence imaging (BLI). The resulting cell line (MDA‐MB‐231‐luc) was next lentivirally transduced with either dCas9‐KRAB All gRNA or with No gRNA, and the transduced cells (2 × 10^6^ cells) were implanted subcutaneously in nude BALB/c mice (*n* = 15 per group). Tumor growth was assessed by caliper measurement and with bioluminescence imaging at various time points (**Figure** **3**A), and 3 tumor samples were harvested for histological examination at day 32 (early time‐point) and day 43 (late time‐point). At day 55 post‐implantation (experimental end‐point), at least 3 tumor samples were harvested for each experimental condition.

We observed significant tumor growth reduction promoted by dCas9‐KRAB All gRNA relative to that of No gRNA and MDA‐MB‐231‐luc (wild type) control cell lines at all experimental time‐points including days 4, 7, 14, 18, 21, 28, 32 until day 35. Notably, at day 21 post‐implantation, All gRNA induced significantly smaller tumors (111.2 ± 10.13 mm^3^, *p* = 0.0089) than No gRNA (164.3 ± 15.62 mm^3^) and the MDA‐MB‐231‐luc wild type line (160.9 ± 58.2 mm^3^) (Figure 3B). Reduction of tumor growth was similarly maintained at day 32 with tumors for All gRNA (174.8 ± 32.1 mm^3^, *p* = 0.0170) relative to No gRNA (243.8 ± 20.3 mm^3^) and the MDA‐MB‐231‐luc wild type line (255 ± 30.7 mm^3^). Consistently, the bioluminescent intensity was also significantly lower in All gRNA at days 21 and day 32 as compared to no gRNA (*p* = 0.0011 and *p* = 0.0449, respectively) Figure 3C. Tumor volumes and bioluminescent intensity plots for mice across all experimental time‐points are compiled in Figure S5A,B, Supporting Information. A description of histological examination is provided in Table S2, Supporting Information, inclusive of IHC expression intensities and % positive cells for CK19 and VIM. Notably comparing summated % expression levels, CK19 expressions rose for All gRNA compared to No gRNA for both early (30 vs 15%) and late (33 vs 16%) collection time‐points. In contrast, VIM expressions fell, particularly at the invasive tumor edge, for dCas9‐KRAB All gRNA compared to No RNA at early (29 vs 46%) and late (23 vs 59%) collection time‐points, in keeping with a mesenchymal to epithelial transition. While co‐staining of CK19 and VIM in the same cell was rare (<10%), we scored small numerical increases with All RNA compared to No gRNA, suggesting a hybrid‐like phenotype (Figure S7A, Supporting Information). These data that was congruent with the in vitro studies carried in transduced cell lines (Figure S4, Supporting Information). Similarly to that observed in in vitro studies, E‐cad was not detected in the All gRNA extracted tumors (Figure S6, Supporting Information). As expected, increased necrosis was observed in the All gRNA tumors compared to controls at early collection time‐points (35 vs 27%).

**Figure 4:**
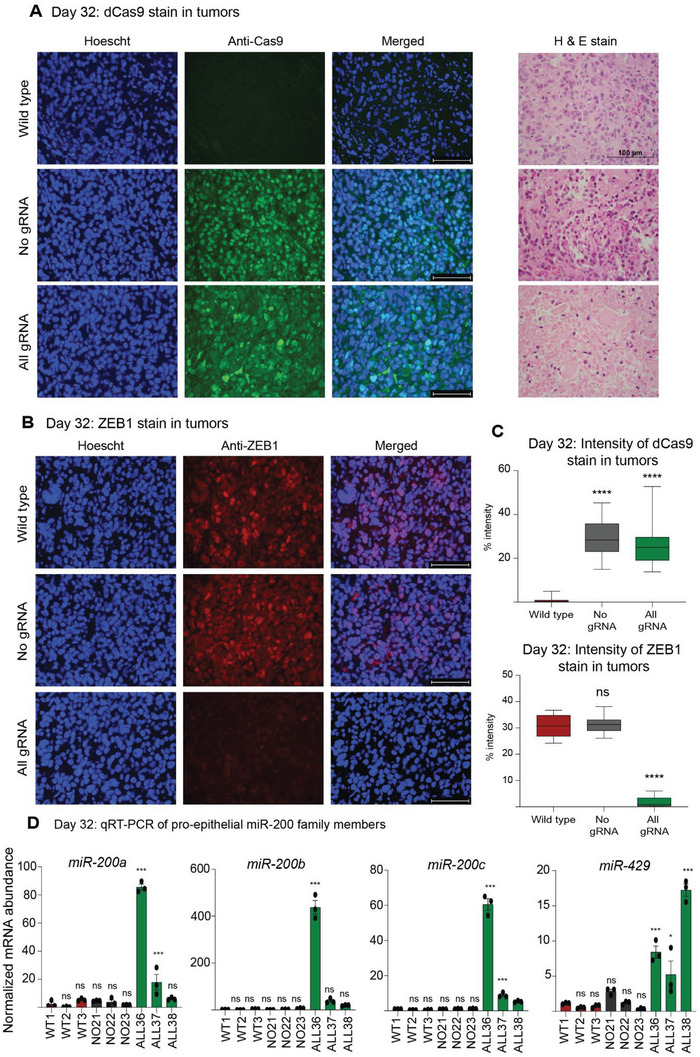
CRISPR‐dCas9‐KRAB induces sustained silencing of ZEB1 in vivo. Representative images of the resected dCas9‐KRAB‐edited tumors versus controls (Figure 3) by immunofluorescence for the detection of (A) dCas9 (*green*) and (B) ZEB1 (*red*). MDA‐MB‐231‐luc tumors either untransduced (Wild type) or transduced with dCas9‐KRAB in absence of gRNA (No gRNA), or expressing All gRNA were analyzed at day 32 post‐implantation of the cells. Hematoxylin & Eosin (H&E) staining of serial sections is indicated to illustrate cellularity. (C) Box and whisker plot highlighting the percent staining intensity of dCas9 and ZEB1 in tumors extract at day 32 relative to untransduced (Wild type). (D) Gene expression analyses by qRT‐PCR to assess the regulation of the pro‐epithelial miR‐200 family members in vivo, with tumors resected at day 32 post‐implantation. ns = non‐significant, *p ≤ 0.05, **p ≤ 0.01, ***p ≤ 0.001 and ****p ≤ 0.0001; miR: microRNA.

RNA abundance analyses by qRT‐PCR in the resected tumors (*n* = 3) collected at day 32 demonstrated significant down‐regulation of *ZEB1* (down by 0.66‐, 0.74‐, and 0.81‐fold in each of the 3 individual specimens, *p*‐values < 0.0001) accompanied by an up‐regulation of *CDH1* (up 34.26, 5.7, and 10.57‐fold respectively, *p*‐values < 0.001) when comparing the All gRNA relative to wild type and No gRNA tumors, suggesting that dCas9‐KRAB effectively maintained the silencing of *ZEB1* with long‐lasting effects (Figure 3D). In addition, expression profiling by qRT‐PCR was performed at day 43 (*n = 3*) and importantly, at endpoint 55 (*n = 4*), where both sets of tumors displayed significant down‐regulation of *ZEB1* and concomitant increases on epithelial markers, such as *CDH1* (Figure 3D). Histological examination of the All gRNA tumors at day 32 validated both the expression of dCas9‐KRAB (**Figure** **4**A) and the maintenance of the down‐regulation of ZEB1 at protein level (Figure 4B). The intensity of the ZEB1 staining was significantly decreased in All gRNA relative to wild type and No gRNA tumors (*p*‐values < 0.0001, Figure 4C). As expected, we observed sustained expression of dCas9 in both All gRNA and No gRNA relative to that of wild type tumors (*p*‐values < 0.0001, Figure 4C). Interestingly, the suppression of ZEB1 was sufficient to upregulate the expression of the three members of the pro‐epithelial miR‐200 family (miR‐200a, miR‐200c, and miR‐429, *p*‐values < 0.01), consistent with a gain of epithelial features (Figure 4D).

Additionally, gene expression profiling by qRT‐PCR on dCas9‐KRAB All gRNA‐edited tumors revealed up‐regulation of pro‐mesenchymal TFs *SNAI2*, *ZEB2*, and *TWIST1* at day 32 which could explain why the edited tumors were increasing tumor growth after day 32 post‐implantation despite the stability of *ZEB1* silencing induced by dCas9‐KRAB (Figure S7B, Supporting Information). Similarly, we observed significant upregulation of *SNAI2* in all mice on day 43 as well as upregulation of *ZEB2*, *SNAI1*, *TWIST1*, and *TWIST2* on day 55 post‐implantation (Figure S7C,D, Supporting Information).

### 2.4 ZEB1 Repression in Triple‐Negative Breast Cancer Lines Induces a Clinically‐Relevant Hybrid‐Like EMT State

**Figure 5:**
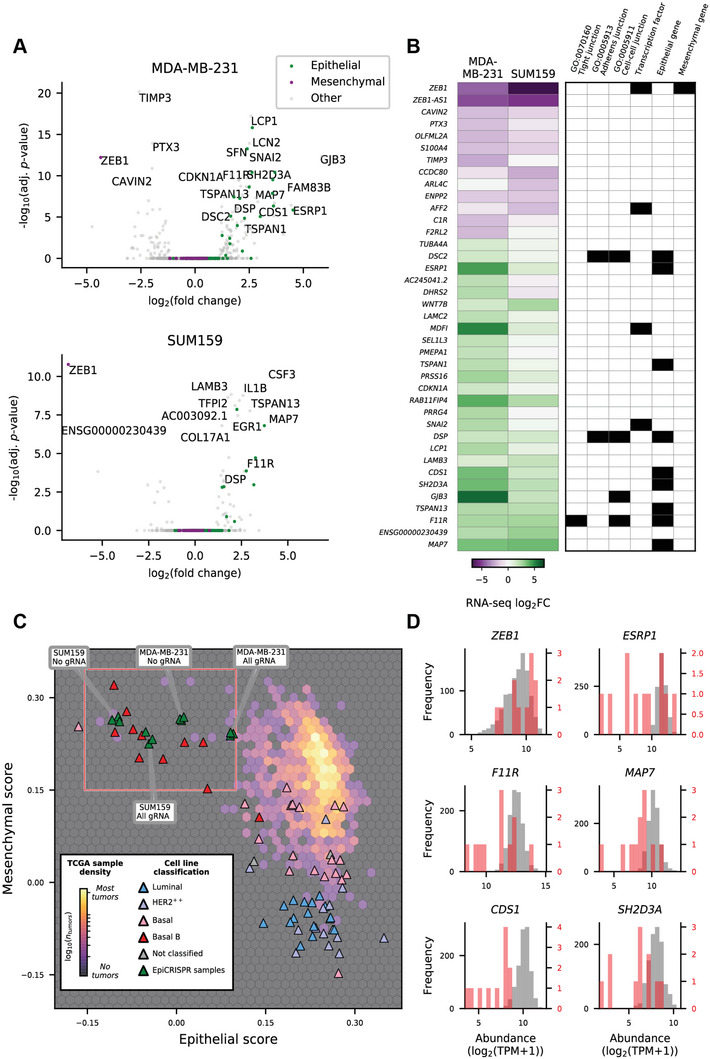
ZEB1 silencing by dCas9‐KRAB induces partial mesenchymal‐to‐epithelial transition. (A) Volcano plots showing changes in transcript abundance assessed by bulk RNA‐sequencing; statistical significance for differential expression of genes between the All gRNA treatment and No gRNA for MDA‐MB‐231 cells (*at top*) and SUM159 cells (*at bottom*). Genes annotated as epithelial or mesenchymal by Tan et al. (2014) are shown. (B) A heatmap showing changes in transcript abundance for genes with significant differential expression (adj. *p*‐value < *n*) between the All gRNA treatment and No gRNA for MDA‐MB‐231 cells or SUM159 cells (*at left*), together with associated Gene Ontology annotations, or Tan et al. (2014) classification as an epithelial or mesenchymal gene (*at right*). (C) A hexbin density plot showing the distribution of TCGA tumor samples when scored with epithelial or mesenchymal gene sets, overlaid with SUM159 and MDA‐MB‐231 RNA‐seq data from this study and CCLE breast cancer cell lines that have been annotated by their subtype classification. (D) Histograms showing the transcript abundance (logTPM) of selected genes (*plot titles*) within TCGA tumor samples with a relatively high mesenchymal score (<0.15) and low epithelial score (<0.1); corresponding to rare claudin‐low/metaplastic tumors (*in red*) and all other TCGA tumor samples (*in gray*). adj. *p*‐value: adjusted *p*‐value, log_2_FC: log 2 fold‐change, Log_2_(TPM): log_2_ of the transcript count per million. Her2+: human epidermal growth factor receptor 2 positive breast cancer, Basal: Basal‐like breast cancer, Luminal: Luminal breast cancers, TCGA: The Cancer Genome Atlas.

The genomic effects of dCas9‐KRAB‐mediated repression of *ZEB1* in the transcriptome of the TNBC cells was first investigated by RNA‐sequencing (RNA‐seq) by comparing differentially and significantly overexpressed transcripts between the gRNA 4 and All gRNA pool relative to No gRNA and untransduced wild type SUM159 and MDA‐MB‐231 control groups. As expected, we observed strong reductions in *ZEB1* transcript abundance upon dCas9‐KRAB editing of *ZEB1* in both cell lines (**Figure** **5**A), confirming the qRT‐PCR data above.

Consistent with the role of *ZEB1* as an epigenetic repressor of epithelial genes, *ZEB1* loss led to up‐regulation of numerous genes implicated in epithelial cell biology, including a number of epithelial marker genes from Tan et al. (2014),^[^ ^41^ ^]^ and significant changes in canonical mesenchymal genes (Figure 5A; *green/purple markers*). Focusing on a subset of genes that showed strong and significant transcriptional changes in at least one cell line, the striking downregulation of *ZEB1* and the associated antisense gene *ZEB1‐AS1* (Figure 5B; *at top*) was apparent. While very few of the other down‐regulated targets have traditionally been associated with mesenchymal phenotypes, as noted above many of the consistently upregulated genes between our cell line models have previously been annotated as associated with an epithelial phenotype (Figure 5B; *at right*). Furthermore, the upregulated genes *F11R* (F11 receptor; previously junctional adhesion molecule 1 [*JAM1*]), *GJB3* (Gap Junction Protein Beta 3), *DSP* (Desmoplakin), and *DSC2* (Desmocollin 2), carry intercellular adhesion related Gene Ontology (GO) annotations, reflecting the induction of these cell functions consistent with a mesenchymal‐to‐epithelial shift.

To investigate the degree to which *ZEB1* repression drove MET in the TNBC cells we explored the relative changes in epithelial and mesenchymal gene set scores in comparison to breast cancer cell lines from the Cancer Cell Line Encyclopedia (CCLE) and primary breast cancer samples from The Cancer Genome Atlas (TCGA) (Figure 5C).^[^ ^39^ ^]^ As expected, these highly‐mesenchymal TNBC cell lines clustered together with other basal B cell lines (*dark red markers*) and a rare subset of metaplastic/claudin low breast cancer tumors (*background hexbin within red box*). Interestingly, despite *ZEB1* repression driving appreciable increases in epithelial score, with the ≈0.12 increase for MDA‐MB‐231 cells corresponding to an average percentile rank increase of 12% for the Tan et al. (2014) epithelial gene list,^[^ ^41^ ^]^ there were only minor reductions in the mesenchymal scores for our model cell lines. This resulted in shifting the samples toward the basal subtype cell lines (*pink markers*) which cluster toward the top right with relatively high epithelial and mesenchymal scores – a feature seen for the majority of TCGA primary tumor samples. Interestingly, several of the pro‐epithelial genes controlled by the ZEB1 regulatory network also show large differences in transcript abundance for related clinical samples, such as *ESRP1*, *F11R*, *MAP7*, *CDS1*, and *SH2D3A* (red box in Figure 5C; histograms in Figure 5D). This observation indicates that our cell line model for *ZEB1* modulation may provide a model with direct relevance for clinical claudin‐low/metaplastic TNBCs. Expression levels of a panel of additional transcription factors associated with EMT^[^ ^61^ ^]^ were also assessed first by qRT‐PCR and RNA‐seq in SUM159 and MDA‐MB‐231 cells, including *OVOL1/2*, *GRHL2*, and *KLF4* (Figure S8A,B, Supporting Information). Highlighting the targeting specificity of this dCas9‐KRAB system, ZEB1 was the only EMT‐related TF gene to show consistently downregulated transcript abundance in both models, however we note for discussion below that *SNAI2* (SLUG) showed significant upregulation within the MDA‐MB‐231 line, and while it was not significantly upregulated within SUM159 cells, this cell line has higher SNAI2 transcript abundance within WT cells relative to MDA MB 231 cells (Figure S8B, Supporting Information).

**Figure 6:**
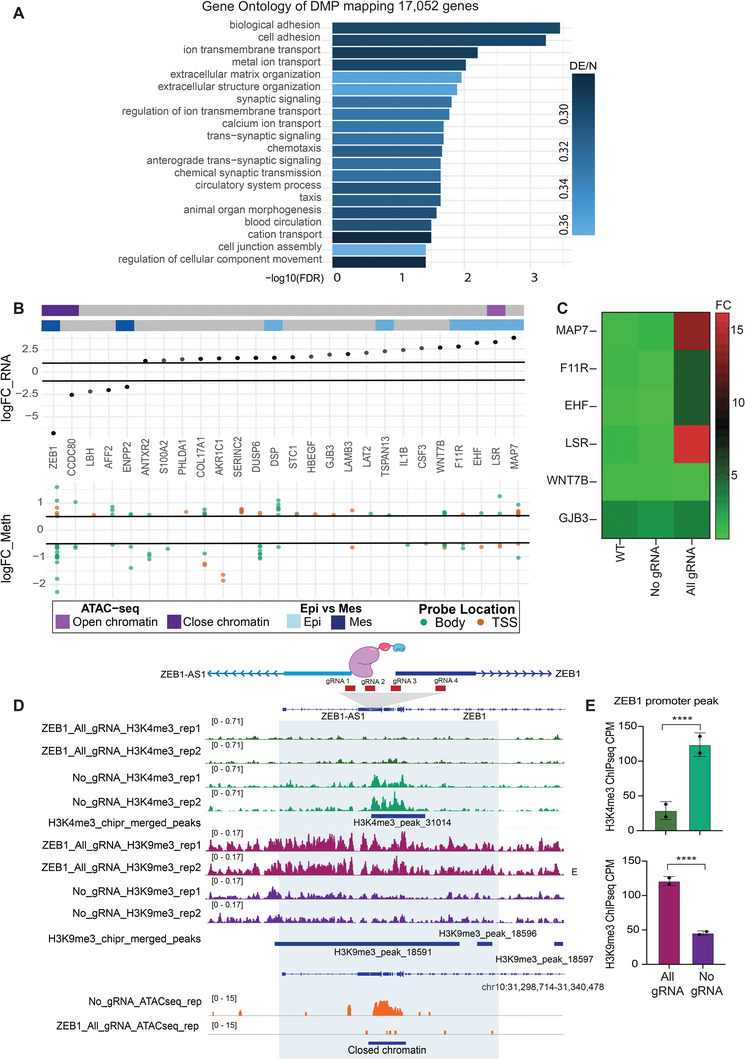
dCas9‐KRAB‐mediated silencing of ZEB1 reprograms the transcriptional and epigenetic state of mesenchymal breast cancer cells. (A) Significance (*‐log_10_(FDR)*) for the top biological terms obtained from Gene Ontology analysis using 87 638 differentially methylated probes mapping to 17 052 genes in SUM159. Bars are colored by the proportion of differentially methylated probes relative to the total number of probes associated with this gene set (DE/N). Several are enriched in cell–cell interaction and cell adhesion suggesting a role in MET. (B) Changes in transcript abundance (log_2_(fold change); *logFC_RNA*) and probe‐level changes in methylation (*logFC_Meth*) for 26 genes that show differential transcript abundance between the All gRNA treatment and No gRNA in SUM159 cells, which also have at least one differentially‐methylated probe. Genes are annotated with chromatin accessibility (ATAC‐seq) data and whether or not they are known EMT markers (*at left*), and are ordered based on RNA‐seq logFC. Probes scatter markers are colored by TSS (*orange*) or gene‐body (*green*) annotation. (C) Transcript abundance for selected genes within treated MDA‐MB‐231‐luc tumor samples (*condition at bottom*) as measured by qRT‐PCR, normalized relative to untransduced wild type tumor samples. (D) Native chromatin immunoprecipitation (ChIP) and assay of transposase accessible chromatin (ATAC) sequencing identifying epigenetic changes at the ZEB1 promoter of All gRNA and No gRNA SUM159 cells. (E) ChIP seq Counts per million (CPM) mapped reads significance in H3K4me3 and H3K9me3 at the ZEB1 promoter peak where ****p ≤ 0.0001. LogFC: log fold‐change, FC: fold‐change, WT: wild type untransduced sample, Epi: epithelial, Mes: mesenchymal, TSS: transcriptional start site, rep: replicate, H3K4me3: histone H3 trimethylation at fourth lysine residue, H3K9me3: histone H3 trimethylation at ninth lysine residue.

Given the gain of epithelial characteristics observed with RNA‐seq, and previous observations that distinct epigenetic signatures are associated with mesenchymal and epithelial cell phenotypes as well as the distinct subtypes of breast cancers,^[^ ^62^ ^]^ we next interrogated whether *ZEB1* silencing induced changes in DNAme. To this end, the same CRISPR‐edited samples and controls were processed using 850K DNAme arrays. Remarkably, there were 87 638 differentially methylated probes (adj *p‐*value < 0.05 and absolute logFC > 0.5) mapping to 17 052 genes. Further detailing the location of each differentially methylated probes; 15 569 mapped to transcription start sites (within 1,500 bp) and 31 032 mapped to gene bodies. Remaining probes were mapped within the first exon (2042), 3′ UTR (1844), 5′UTR (7102), and exon boundary (431) of associated genes. Consistent with the observed gain of epithelial phenotype, Gene Ontology (GO) analyses of differentially methylated genes demonstrated a strong enrichment for cell adhesion, extracellular matrix organization and other EMT associated programs within the differentially methylated probes (**Figure** **6**A).

Focusing on genes that showed strong and significant transcriptional changes in SUM159 (*n*_genes_ = 82, log_2_FC > 1 and adj *p*‐value < 0.05), we found 26 of these genes had also differentially methylated probes based on the methylation data on this cell line (*n*_probes_ = 127, using adj *p*‐value < 0.05 and absolute logFC > 0.5 as thresholds, Figure 6B). These include genes involved in cell adhesion (*F11R*, *COL17A1*, *CCDC80*, *LAMB3*, *DSP*, *MAP7*), lipid metabolism (*LSR*) and immune responses (*CSF3*, *IL1B*). Additionally 26 probes had significant changes in methylation with 6 probes associated with *ZEB1* (Figure S9, Supporting Information). Two of the 6 probes captured changes in methylation within the TSS, while there were 3 probes in the gene body and one in the 3′ UTR.

Amongst the genes which showed a significant increase in transcript abundance following *ZEB1* repression in the SUM159 cell line, the gene *LSR*, which has been annotated as strongly associated with an epithelial phenotype by Tan et al. (2014),^[^ ^41^ ^]^ also had a shift toward open and more accessible chromatin, as assessed by ATAC‐seq data (Figure 6B, *at top, light purple*). Transcriptional down‐regulation of *ZEB1* and *CCDC80* was also accompanied by decreased chromatin accessibility (Figure 6B, *at top, dark purple*). While there was some trend toward transcriptionally up‐regulated genes showing a decrease in gene body methylation, and vice versa, there does not appear to be any definitive association between changes in RNA transcript abundance and gene body methylation (Figure 6B). Subsequent RNA expression analyses from resected MDA‐MB‐231‐luc tumors treated with dCas9‐KRAB All gRNA versus No gRNA (*n = 3* mice per group, day 32 post‐implantation) also confirmed regulation of multiple target genes found on the 26‐gene signature of targets differentially expressed and methylated, notably the up‐regulation of *MAP7*, *F11R*, *EHF*, and *LSR* (Figure 6C).

To investigate what epigenetic changes are being driven by dCas9‐KRAB‐mediated repression of *ZEB1*, we performed ATAC‐seq and native ChIP‐seq for H3K9me3 and H3K4me3 (Figure 6D). Reduced chromatin accessibility was observed at the *ZEB1* promoter in the dCas9‐KRAB All gRNA condition. There was no difference between the fragment count and fragment length (Figure S10A, Supporting Information) and UpSet plots were used to visualize and intersect overlapping peaks between the All gRNA and No gRNA conditions (Figure S10B, Supporting Information). In addition to a decrease in chromatin accessibility in the *ZEB1* locus, we observed significant removal of H3K4me3 concomitant with induction of H3K9me3 (Figure 6D–E). Thus, targeting dCas9‐KRAB to the *ZEB1* promoter led to a remarkable reduction of H3K4me3, a hallmark of active promoters.^[^ ^63^ ^]^ Deposition of H3K9me3 was widespread, spanning ≈21 602 base pairs flanking gRNA target sites within the promoter region, and overlapping with *ZEB1‐AS*, an oncogenic long non‐coding RNA that interacts with lysine methyltransferase KMT2A to epigenetically upregulate *ZEB1*.^[^ ^64^ ^]^ The ChIP‐seq data revealed significant changes (adj. *p*‐value < 0.05) in H3K9me3 associated with 15 genes (Table S3, Supporting Information), including the pro‐epithelial genes *LAMA3*, *PCDHB5* and *CTNNA3* which play crucial roles in remodeling cell adhesion.

### 2.5 A Signature of Differentially Expressed and Methylated Targets upon ZEB1 Silencing Discriminates Breast Cancer Subtypes and is Predictive of Prognosis

**Figure 7:**
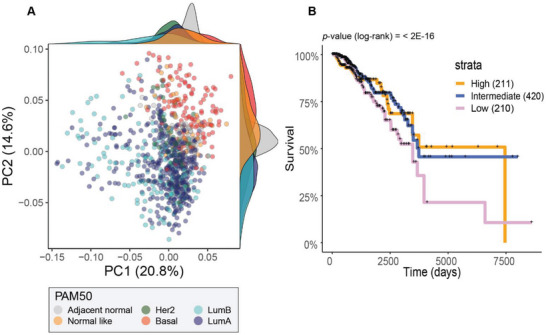
TCGA patient data show subtype‐dependent differences in DNAme at genes identified in our cell line models and a survival association with the relative expression of these genes. (A) Principal component plot using probes from the TCGA‐BRCA 450k DNAme data that were common with differentially methylated 850k probes identified between All gRNA and No gRNA SUM159 samples. Scatter markers are colored by PAM50‐defined subtype and the marginal distributions of PC1 and PC2 scores are shown for each subtype (*kernel density plots at top and at right*). (B) TCGA breast cancer tumors were scored according to the relative abundance of these genes and split into tumors with a high (top 25%), medium (25^th^ – 7^th^ percentile) or low (bottom 25%) score for generation of Kaplan–Meier (KM) survival curves. Significant differences in survival were assessed by a KM log‐rank test. TCGA‐BRCA: The Cancer Genome Atlas Breast Invasive Carcinoma, DNAme: DNA methylation, PC: principal component, Her2: human epidermal growth factor receptor 2 positive breast cancers, Basal: Basal‐like breast cancers, LumB: Luminal B breast cancers, LumA: Luminal A breast cancers.

Given the ability of *ZEB1* silencing to drive transcriptional changes with evidence of epigenetic reprogramming, we next investigated the clinical relevance of the associated DNAme changes for these 26 genes across clinical breast cancer tumor samples. Using *β*‐value DNAme data from the TCGA we extracted probe‐level data for all genes listed in Figure 6B and performed a principal component (PC) analysis. As shown, basal‐like breast cancer samples (as defined by PAM50 annotations) clearly separate from luminal subtypes across the first two principal components (**Figure** **7**A) demonstrating that these genes show large variations in DNAme across the cancer subtypes (which also show large differences in epithelial‐mesenchymal characteristics; Figure 5c). To further investigate how the DNAme at these genes varies with EMT, we examined the associations between probe‐level *β*‐values and epithelial score or mesenchymal score, with markers colored by subtype. When examining the Spearman's correlations between the probe‐level methylation and bulk tumor epithelial score and mesenchymal score, a strong negative association was observed, such that probes positively‐correlated with the epithelial score tended to have a negative correlation with the mesenchymal score (and vice versa; Figure S11, Supporting Information). Furthermore, when the genes differentially expressed (both upregulated and downregulated differential expression) in our SUM159 cell line model were used for gene‐set scoring, patients carrying tumors with a “low” score (bottom 25% Figure 7A) had a significantly worse overall survival (*p*‐value < 2 × 10^−16^; KM log‐rank test comparing Low versus Medium/High groups; Figure 7B), highlighting a potential role for the ZEB1 regulatory program in clinical patient outcomes.

**Figure 8:**
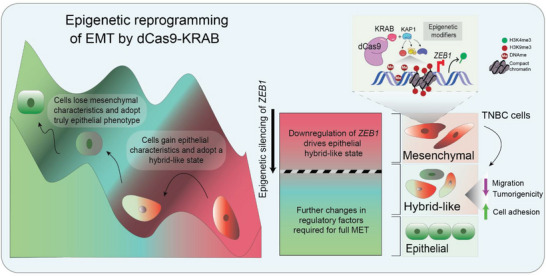
Schematic model illustrating the effects of dCas9‐KRAB silencing ZEB1 in reprogramming epithelial plasticity. Following epigenetic silencing of ZEB1 by dCas9‐KRAB, TNBC cells acquire epithelial characteristics shifting with a resemblance to hybrid‐like states that might be present in human breast cancer. To fully drive the cells to a luminal lineage would require targeting of multiple factors, such as other EMT‐TFs, to overcome epigenetic barriers of mesenchymal cells. EMT: Epithelial to mesenchymal transition, MET: Mesenchymal to epithelial transition, KRAB: Krüppel associated box, DNAme: DNA methylation, H3K4me3: histone H3 trimethylation at fourth lysine residue, H3K9me3: histone H3 trimethylation at nineth lysine residue.

Our model suggests that *ZEB1* silencing by dCas9 systems reprogram the epigenetic landscape of TNBCs toward a more epithelial phenotype that most resembles the hybrid‐like states present in human breast tumors. Despite the gain in epithelial features induced by *ZEB1* silencing, these cells were not completely shifted to the luminal lineage, suggesting that ZEB1 silencing was not sufficient to overcome the epigenetic barriers of mesenchymal cells, and that additional factors might be necessary to target (including possibly other EMT‐TFs) to achieve a more robust completion of MET (**Figure** **8**). Our model suggests that dCas9‐KRAB mediated silencing of *ZEB1* of in mesenchymal TNBCs, for example, MDA‐MB‐231 cells also resulted in enhanced cell adhesion, impaired cell migration, and inhibition of tumorigenesis in vivo. Moreover, integrated analyses of the transcriptional and epigenetic changes induced by dCas9‐KRAB identified a gene set associated with EMP programs, including cell adhesion and migration, which was also predictive of breast cancer patient's outcome.

## 3 Discussion

Herein, for the first time we show that CRISPR/dCas9 technology adapted for epigenetic silencing can epigenetically reprogram cancerous TNBC cells toward a more epithelial, less migratory and less tumorigenic phenotype, opening the door to synthetic strategies to shift aggressive TNBC cell populations toward potentially more luminal and benign counterparts in a clinical setting. These results have important implications, both for the development of novel personalized treatments and precision medicine approaches for aggressive BCs, and in providing previously unexplored experimental model systems to understand epithelial‐mesenchymal plasticity.

Consistent with previous literature, all the designed gRNAs targeted *ZEB1* with high potency and selectivity, with negligible off‐target activities.^[^ ^25^ ^]^ The epigenetic editing induced heterochromatin formation centered on the 4 gRNA‐binding sites, as reflected by induction of H3K9me3, induction of methylation at specific CpG sites, and formation of less accessible chromatin. Interestingly, the dCas9‐KRAB nucleated heterochromatin formation extended locally, with ≈9000 bp downstream of the *ZEB1* locus and ≈12 000 bp upstream of the *ZEB1* locus, into the promoter and coding region of the neighboring gene, *ZEB1 antisense 1* (*ZEB1‐AS1*), which encodes a long‐non coding RNA that positively regulates the expression of *ZEB1* in hepatocellular carcinoma^[^ ^65^ ^]^ and prostate cancer models.^[^ ^64^ ^]^ Thus, it is possible that the formation of repressive heterochromatin at this locus could also re‐enforce *ZEB1* silencing catalyzed by dCas9‐KRAB in the edited TNBC cells.

Previous studies have investigated the loss‐of‐function of ZEB1 by Cas9‐mediated ZEB1 knockouts,^[^ ^66^, ^67^ ^]^ shRNA,^[^ ^68^, ^69^ ^]^ and siRNA^[^ ^70^ ^]^ and by gene knockout studies in mice.^[^ ^71^ ^]^ Whilst these reports also re‐enforced the key role of ZEB1 in remodeling cell adhesion, and inhibiting migration and cell growth in several cancers, these studies did not report epigenetic reprogramming. In this context, advantages of dCas9‐KRAB systems relative to RNA interference technologies include the induction of programmed epigenetic editing in the genome, leading to potentially long‐lasting epigenetic repression at targeted sites. Interestingly, the epigenetic silencing of ZEB1 did not affect cellular proliferation nor apoptosis in vitro and in vivo, as assessed by Ki‐67 and cleaved caspase 3 assays (Figure S12, Supporting Information), but negatively impacted anchorage independence in vitro and tumorigenicity in vivo. In other systems such as siRNA and shRNA, it has been shown that EMT results in growth arrest via cell cycle regulation, for example, via Cyclin D1/2.^[^ ^72^, ^73^, ^74^ ^]^ The lack of regulation of proliferative/cell cycle genes in our RNA‐seq studies could be again associated with partial MET phenotype triggered with ZEB1 silencing, which resulted mostly in enrichment of cellular adhesion molecules.

Previous reports have also outlined the localized spread of H3K9me3 by dCas9‐KRAB methods,^[^ ^75^, ^76^ ^]^ such as in the HS2 enhancer in the *globin* control locus region in human cells,^[^ ^25^ ^]^ and KRAB‐containing zinc finger proteins have demonstrated potential to mediate long‐range heterochromatin spreading.^[^ ^77^ ^]^ In our hands, most synthetic zinc finger proteins linked to KRAB failed to induce DNAme, requiring the engineering with DNMT3A/3L to write DNAme.^[^ ^78^, ^79^, ^80^ ^]^ The observed induction of H3K9me3 by dCas9‐KRAB in the *ZEB1* locus is likely mediated by the recruitment of lysine 9‐specific methyltransferase SETDB1^[^ ^57^ ^]^ via the KRAB‐associated protein 1 (KAP1) co‐repressor. Similarly, tri‐methylation of H3K9 can act as a binding scaffold for Heterochromatin protein 1 alpha (HP1*α*), which compacts and maintains condensed chromatin structure.^[^ ^81^ ^]^ In addition, the recruitment of the NuRD complex by the KRAB domain can influence the subsequent recruitment of DNMTs and/or methyl‐binding proteins, such as MeCP1.^[^ ^82^, ^83^ ^]^ Last, the ability of DNMT3A to predominantly recognize unmethylated H3K4^[^ ^84^ ^]^ could contribute to the changes in DNAme in the *ZEB1* promoter.

Interestingly, we observed significant removal of H3K4me3 at the *ZEB1* promoter of dCas9‐KRAB edited cells. To our knowledge, there are no previous reports demonstrating that KRAB directly recruits enzymes capable of erasing H3K4me3. However, this effect could be indirect and due to different molecular cross‐talks between epigenetic modifiers, notably the interaction of H3K4me3 demethylase (KDM5C/JARID1C) at sites containing H3K9me3 via its “plant homeodomain” (PHD) finger.^[^ ^85^ ^]^ Thus, the local spread of H3K9me3 may have prompted the recruitment of JARID1C or other histone demethylases such as JARID1A (KDM5A/RBP2), JARID1B (KDM5B/PLU‐1) and/or JARID1D (KDM5D/SMCY), all of which can remove H3K4me3.^[^ ^85^ ^]^ Finally, it has been reported that the *ZEB1* promoter is marked with bivalent chromatin modifications, namely, H3K27me3 and H3K4me3, in a “plastic” subpopulation of human basal breast cancers.^[^ ^16^, ^86^ ^]^ Thus, it is also conceivable that this bivalent state could poise the *ZEB1* promoter for effective gene silencing.^[^ ^87^ ^]^ In fact, fusions of dCas9‐Ezh2 (the catalytic subunit of the Polycomb Repressive Complex 2 (PRC2)), inducing targeted H3K27me3, have been shown to promote epigenetic memory in presence of DNMT3A/3L in specific promoters, suggesting that the writing of H3K27me3 could be important for attenuation of gene expression in a promoter and/or context‐dependent manner.^[^ ^88^ ^]^

In contrast with dCas9‐KRAB‐dependent epigenetic changes observed at the *ZEB1* locus, the downstream epigenetic changes or reprogramming observed elsewhere in the genome may be explained by the regulation of *ZEB1* expression, or by modulation of downstream TFs and/or epigenetic modifiers (notably, SETDB1) regulated by ZEB1.^[^ ^70^ ^]^ The complexity of interplay between TFs, such as ZEB1, and epigenetics regulators have been studied in EMT transcriptional networks. A genome‐wide CRISPR screen identified two histone methyl transferases, PRC2 and KMT2D‐COMPASS, as epigenetic barrier to plasticity with knockouts of their subunits EED and ASH2L allowed epithelial cells display phenotypic changes by acquire EMP.^[^ ^89^ ^]^ In the same study, SNAIL expression is affected by PCR2 knockout despite not being a direct target. However, in another study, PCR2 was shown to only affect SNAIL expression by a complex epigenetic interplay involving SNAIL, ZEB1 and PPPX1.^[^ ^90^ ^]^ ZEB1 binds targeted genes that contain E‐boxes consensus sites and regulate these genes by interacting with diverse epigenetic modifiers. One notable interaction occurs between ZEB1 and recruitment of CtBP corepressor to the CtBP‐interacting domain^[^ ^91^ ^]^ and has been reported in ZEB1‐mediated *CDH1* repression. However, ZEB1 can also repress CDH1 independent of CtBP, for example by interaction with BRG1, a SWI/SNF chromatin‐remodeling protein.^[^ ^10^ ^]^ An alternative co‐repressor is BIG1/SMARCA4, and genomic loss of SMARCA4 has been linked to mesenchymal change and therapy response in lung and breast cancers.^[^ ^92^ ^]^ The canonical role of ZEB1 as a suppressor of *CDH1* has been demonstrated in metastatic prostate cancer,^[^ ^93^ ^]^ where it is required for SIRT1 recruitment, mediating deacetylation of histone H3 to block RNAPol recruitment and causing transcriptional repression. In pancreatic cancer, ZEB1 forms a complex with HDAC1 and HDAC2 at the *CDH1* promoter, resulting in down‐regulation of transcript and protein.^[^ ^94^ ^]^ Similarly, in breast cancer, ZEB1 can form a complex with HDAC1/DNMT3B on the *ER‐α* and *Ngn3* promoters, respectively.^[^ ^95^, ^96^ ^]^ So important is the interplay between EMT‐TFs and epigenetic regulators, the complexity of epithelial plasticity is further challenged by a recent study where epigenetic modifiers can govern the duration of EMT reversibility.^[^ ^97^ ^]^ Chromatin accessibility affects transcription and regulation with a direct effect on epithelial plasticity.^[^ ^98^ ^]^ For example, transcription factor, Nuclear factor I (NFI), induces EMT by upregulating expression of SNAIL.^[^ ^99^ ^]^ ATAC‐seq was able to demonstrate that this EMT‐TF was capable of opening chromatin of ≈1800 distal regulatory regions in human small cell lung cancer.^[^ ^100^ ^]^ An elegant study by Cieslik et al. (2013) revealed distinct EMT‐related gene clusters by chromatin profiling. Putative enhancer sites enriched with histone modifications H3K4me1 or H3K27ac where found to be differentially marked between epithelial and mesenchymal states.^[^ ^101^ ^]^

The integration of the transcriptional and epigenetic changes promoted by CRISPR/dCas9‐KRAB in our cell line models enabled the discovery of a set of 26 genes central to the regulatory network controlled by ZEB1, and the transcriptional status of these targets (up and downregulation) in both cell lines is included in Figure S13, Supporting Information. The DNAme status of probes mapping in these genes demonstrated significant variations between PAM50 (transcriptome‐defined) subtypes of BC and carried significant prognostic association with patient overall survival within clinical breast cancer (TCGA) cohort. It is tempting, therefore, to speculate that these changes reflect some of the epigenetic reprogramming that occurs with epithelial mesenchymal plasticity during breast cancer progression in patients. Genes central to cellular polarity and adhesion, including *CDH1* have previously been identified as targets of *ZEB1* in alternative models of breast cancer.^[^ ^17^ ^]^ *Microtubule associated protein 7* (*MAP7*) has been previously implicated in intercellular adhesion of differentiating keratinocytes,^[^ ^102^ ^]^ and similarly the laminin‐5 subunits *LAMB3* and *LAMC2* are critical for the epithelial tissue basement membrane and associated epithelial tissue‐level polarization.^[^ ^103^ ^]^ Interestingly, very few canonical mesenchymal genes showed significantly changes in the SUM159 or MDA‐MB‐231 cell line. However, Caveolae Associated Protein 1 (*CAVIN1*/*PTRF*) is a mesenchymal cell line gene identified by Tan et al.,^[^ ^41^ ^]^ and *CAVIN2* showed strong and consistent down‐regulation in both of our cell line models. Further, the inflammatory cytokine pentraxin 3 (*PTX3*) has been observed to change in poorly‐differentiated clinical breast cancer samples^[^ ^104^ ^]^ and the S100 calcium binding family member *S100A4* has been implicated in endometrial cancer progression through EMT.^[^ ^105^ ^]^

Our model suggests that targeted epigenetic silencing of *ZEB1* is sufficient to induce an epigenetic landscape that resembles the hybrid‐like states present in human breast tumors. While these cells significantly gained epithelial features, they were not shifted completely to the luminal lineage, with only modest variations in the mesenchymal score. This suggests that *ZEB1* silencing was not sufficient to overcome epigenetic barriers of mesenchymal cells, and consequently simultaneous targeting of multiple other EMT regulators might be necessary to complete and reverse EMT. Notably in sarcomas, the combinatorial overexpression of GRHL2, downregulation of ZEB1 and/or over expression of miR‐200 was required to synergistically upregulate epithelial genes, thus inducing a MET‐like phenotype.^[^ ^106^ ^]^ Our work reinforces the concept that the ZEB1/miR‐200 regulatory loop control the transition between the Mesenchymal‐hybrid (mesenchymal and epithelial) states, and interestingly, epigenetic silencing of *ZEB1* was sufficient to upregulate all the individual miR‐200 cluster members. Both ZEB1 and SLUG have been shown to be highly expressed in mesenchymal cells, while TWIST1/2 and SNAIL levels were significantly increased in hybrid populations.^[^ ^107^ ^]^ Consequently it is plausible that co‐silencing of ZEB1 with that of TWIST1/2 and SNAI1/2 factors could further facilitate the loss of mesenchymal scores. As the plasticity of hybrid‐like states in human breast cancers has been correlated with metastatic potential,^[^ ^5^ ^]^ co‐suppression of multiple EMT factors via multiplexing CRISPR methods represents an important step toward a more efficient and robust reprograming of TNBCs.

Further supporting this notion, our in vitro and in vivo analyses demonstrate that silencing of *ZEB1* resulted in up‐regulation of SLUG. This could explain the therapeutic escape of MDA‐MB‐231 tumors despite the stability or maintenance of the *ZEB1* silencing in vivo. Mathematical modeling of the ZEB1 regulatory circuit indicated that SNAI2 (SLUG) may play a role in blocking complete an EMT, which is consistent with the results obtained in this study.^[^ ^108^ ^]^ There are multiple evidence that EMT‐TFs can regulate each other, and in particular that SLUG, and SNAIL and TWIST1 control ZEB1 expression.^[^ ^109^, ^110^ ^]^ Thus, blockade of more than one EMT‐TFs might be required to completely suppress the underlying molecular mechanisms of EMT‐TF transcriptional cross‐talks, potentially leading to therapeutic escape. The plasticity of hybrid‐like states in human breast cancers has been correlated with metastatic potential.^[^ ^5^ ^]^ Interestingly our data suggest that synthetic silencing of ZEB1 results on decreased tumorigenesis and inhibition of cellular migration. Furthermore, our bioinformatics analyses also suggest the emergence of a hybrid‐like state associated with better prognosis in breast cancer patients. However, future works should include the impact of down‐regulation of EMT‐TFs on metastatic colonization and macrometastasis formation, as well as resistance to chemotherapies. Our data suggest that downregulation of additional inhibition factors including but not limited to SLUG, SNAIL, TWIST1, and ZEB1 could be required to drive the cells to an epithelial lineage. Excitingly, our data suggest that reverse reprograming of EMT might be possible by targeted epigenetic silencing of EMT‐TFs and that synthetic biology/precision medicine approaches can be harnessed to model and target potentially the multiple hybrid and epigenetic states relevant to many highly aggressive cancers such as bladder and lung cancers.

## 4 Experimental Section

### Guide Design and Cloning

CRISPR/dCas9 fused to the effector domain KRAB was selected for repression of *ZEB1*. The pLV hU6‐sgRNA hUbC‐dCas9‐KRAB‐T2a‐Puro (Addgene #71236) was a third‐generation plasmid that contains a single expression lentiviral vector with sgRNA driven by a U6 promoter and 3*×* FLAG tagged dCas9 with KRAB fused to its C‐terminus.^[^ ^25^ ^]^ From here onward, this will be referred to as pLV dCas9‐KRAB. The pLV hU6‐sgRNA hUbC‐dCas9‐No eff‐T2a‐Puro plasmid was generated from the pLV hU6‐sgRNA hUbC‐dCas9‐KRAB‐T2a‐Puro template by restriction enzyme digestion with NheI (NEB) (flanking both of ends of the KRAB sequence). Following gel purification, the resulting plasmid was reannealed and removal of KRAB confirmed by analytical digest and Sangar Sequencing (AGRF Perth).

A selection of four sgRNAs with optimized on‐target^[^ ^30^ ^]^ and off‐target scores^[^ ^31^ ^]^ (Benchling) were selected in a 400 bp search area in the *ZEB1* proximal promoter. Selected sgRNA target sequences (Integrated DNA Technologies) and their scores are listed in Table S1A, Supporting Information. Guide oligos were cloned into the pLV dCas9‐KRAB vector as previously described.^[^ ^32^, ^33^ ^]^ Insertion of sgRNA into the expression vector was confirmed by an analytical digest and Sanger Sequencing (AGRF Perth). Unless otherwise stated, All gRNA conditions contained equal plasmid concentrations of 4 gRNA.

### Cell Lines

The human embryonic kidney 293T (HEK293T, CRL‐3216) and TNBC MDA‐MB‐231 (HTB26) were obtained from American Type Culture Collection (ATCC, Manassas, VA). HEK293T and MDA‐MB‐231 were cultured in Dulbecco's Modified Eagle's Medium (DMEM) with high glucose‐pyruvate supplemented with heat inactivated fetal bovine serum (FBS) and 1% Antibiotic‐Antimycotic (Anti‐Anti, Gibco). TNBC SUM159 (Asterand Bioscience) were cultured in F12 Medium supplemented with 0.6% 1 m Hepes, 5 µg mL^−1^ insulin, 1 µg mL^−1^ hydrocortisone, 1% Anti‐Anti, and 10% heat inactivated FBS. Unless otherwise stated, all cell lines were grown at 37 °C and 5% CO_2_.

### Lentiviral Transduction Protocol

All lentiviral experiments were performed with Perkins Institutional Biosafety Committee and Office of Gene Technology Regulator Approval #NLRD004/2017 and #NLRD008/2020. Lentiviral particles were produced using HEK293T with co‐transfection using third generation VSVG (Addgene plasmid #12259), GAG/POL (Addgene plasmid #12260) and pLV dCas9‐KRAB +/−gRNA as previously described.^[^ ^26^, ^33^ ^]^ Four days following transduction, 1.25 and 0.75 µg mL^−1^ puromycin was used to select SUM159 and MDA‐MB‐231, respectively. From here on forward, transfected cells are referred to as All gRNA (+4 gRNAs) and No gRNA (‐gRNA). Lentiviral transduction with the pLVX‐IRES‐ZsGreen vector (a kind gift of Prof. Ruth Ganss) was performed to generate MDA‐MB‐231‐luc cell line, for the constitutive expression of the luciferase reporter gene.

### RNA Extraction and RT‐qPCR

In vitro transfected SUM159 and MDA‐MB‐231 cells were harvested, and RNA extractions were performed with QIAzol Lysis Reagent (QIAGEN). RNA from tumor tissue was extracted through disruption and homogenization using a TissueLyser (QIAGEN) then purified using the QIAzol method.

Purified RNA was quality control tested on an agarose gel and Nanodrop (Thermofisher) before using 2 µg of total RNA for cDNA conversion using the High‐Capacity cDNA Reverse Transcription Kit (Applied Biosystems). Relative transcript expression levels were determined using TaqMan probes (Applied Biosystems) in the ViiA 7 Real‐Time PCR machine (Applied Biosystems). Relative quantification of gene expression was normalized to the housekeeping *PPIA*, using the comparative 2^−∆∆Ct^ method.

### Protein Quantification and Western Blot

Protein was extracted from pelleted cells using Lysis Buffer (Cell Signaling Technology). Samples were sonicated for 10 s at 10 mA followed by a 10 min centrifugation at 13 000 rpm, 4 °C. Supernatant was removed and a DC Protein assay (Bio‐Rad) was performed as per manufacturer's protocol. Western blotting was carried out as per protocols previously described.^[^ ^33^ ^]^ Protein samples were run on a Mini‐PROTEAN 10% polyacrylamide gel (Bio‐Rad) and the gel was transferred to PDVF membranes using the Trans‐Blot Turbo Transfer System (Bio‐Rad). Membranes were blocked with agitation in 5% skim milk in TBST (20 mm Tris Base, 150 mm NaCl, 0.1% Tween 20, pH 7.5) and washed before incubating overnight at 4 °C with primary antibodies. The next day, membranes were incubated with corresponding secondary antibodies for 1 h at room temperature (RT). Protein bands were imaged using the ChemiDoc MP Imaging System (Bio‐Rad) and images were processed using the ImageLab Software v5.2 (Bio‐Rad).

### Immunofluorescence assay

UV sterilized glass circular coverslips (10 mm, Thermofisher) were coated with 1:10 Poly‐L‐Lysine. Following incubation, ≈100 000 cells of transfected MDA‐MB‐231 and SUM159 plus wild type were seeded and incubated overnight at 37 °C and 5% CO2. Coverslips were fixed with Pierce 4% Formaldehyde (ThermoFisher), blocked for 1 h at RT (blocking buffer: 5% normal goat serum, 0.3% Triton X‐100 in 1*×* PBS) before being incubated with primary antibodies (1:100, 1% BSA, 1*×* PBS) at 4 °C overnight. The coverslips were then incubated with secondary antibodies Alexa Fluor 488 goat anti‐mouse (1:500, ThermoFisher), Alexa Fluor 594 goat anti‐rabbit (1:500, ThermoFisher), and Hoechst (1:5000, Sigma‐Aldrich) in 1% BSA, 1*×* PBS at RT for 1 h. Coverslips were mounted with SlowFade Diamond Antifade Mountant (Molecular Probes, Eugene, Oregon). Images were captured using a fluorescent microscope (Nikon) and processed using NIS‐Elements Software.

### Soft Agar Colony Formation Assay

Transfected MDA‐MB‐231 and SUM159 plus wild type were tested for colony formation using the soft agar assay using protocols previously described.^[^ ^34^ ^]^ Colonies were left to develop over 4 weeks with a layer of culture medium maintained to prevent desiccation twice weekly. Cells were stained with 0.5 mg mL^−1^ MTT solution in culture medium for 4 h at 37 °C and 5% CO_2_ before imaging and quantification using brightfield microscope IX‐71 (Olympus) and ImageJ software, respectively.

### Migration Assay

Transfected MDA‐MB‐231 and SUM159 plus wild type were subjected to the migration assay to study changes in migratory responses using protocols previously described.^[^ ^33^ ^]^ Cells were starved of FBS for 24 h prior to seeding in media containing 0.05% FBS. For each condition, 3 × 10^4^ cells were seeded into the inner chamber of the Corning Costar Transwell cell culture insert. Medium containing 10% FBS was added as the chemoattractant in the outer chamber. Cells were incubated for 22 h at 37°C and 5% CO_2_. The inner chamber was washed 2*×* in PBS before being stained with a staining solution (0.5% crystal violet, 25% methanol) before being imaged and quantified using brightfield microscope IX‐71 (Olympus) and ImageJ software, respectively. Cell counts were processed under 40*×* magnification with five fields of view per insert.

### RNA‐Sequencing and Analysis

Total RNA was extracted using the QIAzol Lysis Reagent (QIAGEN) as per manufacturer's protocol. RNA libraries were prepared from experimental replicates (individual clones) and sequenced for 50 bp single‐end (SE) reads on the Illumina HiSeq2500 system (AGRF Melbourne). RNA‐sequencing (RNA‐seq) data underwent pseudo‐alignment and quantification with Salmon^[^ ^35^ ^]^ before import into R/Bioconductor using tximport^[^ ^36^ ^]^ then differential expression was performed with voom‐limma^[^ ^37^ ^]^ and the TREAT criteria.^[^ ^38^ ^]^

### Gene Set Scoring

Gene set scoring with breast cancer tumor samples and cell lines was performed as for Cursons et al. (2018).^[^ ^39^ ^]^ Briefly, the python implementation of singscore^[^ ^40^ ^]^ (https://github.com/DavisLaboratory/PySingscore) was applied to Epithelial and Mesenchymal gene sets from Tan et al. (2014).^[^ ^41^ ^]^ RNA‐seq data from The Cancer Genome Atlas (TCGA) Breast Cancer cohort^[^ ^42^ ^]^ (Final RNA data from https://gdc.cancer.gov/about‐data/publications/pancanatlas) were scored for representative tumor samples. Similarly, RNA‐seq data of breast cancer cell lines was obtained from the Cancer Cell Line Encyclopedia (CCLE)^[^ ^43^ ^]^ (22Q2 data from https://depmap.org/portal/download/). To make scores more comparable across these independent data sets only genes present across all studies (*n_genes_* = 16 561) were used for scoring.

### CRISPR gRNA Off‐Target Analysis

Potential *S. pyogenes* CRISPR/Cas9 targets were enumerated in the region 500 nucleotides 5′ of the transcription start site for *ZEB1* to specify on‐target activity for selected gRNAs. Potential off‐target activities for these gRNAs were profiled against the GRCh38 ENSEMBL reference genome, using dsNickFury to implement the Azimuth and Elevation on‐ and off‐target scoring models.^[^ ^30^, ^44^ ^]^ Candidate gRNAs were ranked by their predicted risk of off‐target activity, and the top 4 putative off‐targets for each gRNA were identified for investigation within the RNA‐seq data. Top off target prediction using dsNickFurry are listed in Table S1B, Supporting Information.

### DNA Extraction and Methylation Analysis

Transfected SUM159 cells were harvested before performing DNA extractions with the Monarch Genomic DNA Purification Kit (New England Biolabs) as per manufacturer's protocol. Following quality control, purified DNA was processed using the Illumina Methylation EPIC 850k Array (AGRF Melbourne). Bisulfite reads and methylation data analysis were conducted using the minfi R package to read.idat files into R (version >3.6.3), and the missMethyl R package^[^ ^45^ ^]^ was used for data processing and normalization. Specifically, we identified low quality probes and removed them from the data normalized by SWAN. We then extracted *β* and M‐values (with an offset of 100 for calculating M values). Data quality control was performed using PCA and MDS plots as well as histograms of *β* values, probe‐level differential methylation analysis was performed on M‐values using limma, and probes with an adjusted *p*‐value < 0.05 and absolute log fold change >0.5 were considered as differentially methylated probes (DMPs). To map probes to their associated genes and gene regions, we used the IlluminaHumanMethylationEPICanno.ilm10b2.hg19 R package. Gene Ontology and KEGG pathway analysis was performed using the gometh() function from missMethyl. Data wrangling and visualizations were performed using the tidyr and ggplot2 packages, respectively.

### TCGA Methylation Probe‐Set Analysis and Survival Analysis

DNA methylation (DNAme) data collected for this study used the Infinium MethylationEPIC (850k) array while the TCGA breast cancer data were collected with the Infinium HumanMethylation (450k) arrays. Overlapping 850k probes from the SUM159 model (Figure 5A) that were present with TCGA 450k probes were identified and a principal component analysis was performed using the R svd function. To score TCGA breast cancer samples, up‐ and down‐regulated genes from our SUM159 model were used for gene‐set scoring with singscore.^[^ ^40^ ^]^ After scoring the TCGA tumor samples were split into high (*top* 25%), medium (25^th^ – 75^th^ *percentile*) and low (*bottom* 25%) tumor groups, and the survival outcome of corresponding patients bearing these tumors was assessed by Kaplan–Meier survival curve a Kaplan–Meier log‐rank test using the survival package in R.

### ATAC‐Sequencing and Library Preparation

ATAC‐seq was performed on SUM159 transfected cells and wild type as per the Omni‐ATAC‐seq protocol.^[^ ^46^ ^]^ Briefly, cells were grown to ≈80% confluence in P10 plates. ≈500 000 cells were resuspended and lyzed on ice for 3 min in 50 µL ice‐cold ATAC resuspension buffer (ATAC‐RSB, 10 mm Tris‐HCL pH 7.4, 10 mm NaCl, 3 mm MgCl_2_) containing 0.1% NP40 (Sigma), 0.1% Tween 20 (Sigma), and 0.01% Digitonin (Promega). Following lysis, samples were resuspended in 6 mL ice cold ATAC‐RSB containing 0.1% Tween 20 and pelleted at 4 °C at 500 g for 5 min. Nuclei were resuspended in 50 µL of ice cold ATAC‐RSB before being counted prior to transposition. Tagmentation was performed in 1*×* Tagmentation Buffer (1 m Tris‐HCl pH 7.6, 1 m MgCl2, 10% Dimethyl Formamide) using 2 µL Tn5 transposase (in‐house made Tn5, 25 µg mL^−1^ final) in 100 µL final volume, at 37 °C for 30 min, 1000 rpm. Tagmentation was inactivated with a final concentration of 0.5% SDS (Merck) and subjected to Proteinase K treatment (NEB) at 55 °C for 15 min at 1000 rpm. Reaction was purified using a Bioline PCR Clean‐up kit and eluted in 25 µL of H_2_O. PCR was performed using NEBNext High Fidelity PCR master mix with the following conditions: 58 °C for 5 min, 72 °C for 5 min, 98 °C for 30 sec; 11 cycles of 98 °C for 10 sec, 63 °C for 30 sec, 72 °C for 1 min and then 72 °C for 2 min and hold at 12 °C. Final clean‐up of product was performed using 1.0*×* Ampure XP beads and visualized on the Agilent D5000 TapeStation.

### ATAC‐Seq Data Analysis

ATAC‐seq data were adapter and quality trimmed with fastp^[^ ^47^ ^]^ with the standard settings followed by mapping with bowtie2^[^ ^48^ ^]^ against the human reference genome hg38 in parallel with gnu‐parallel.^[^ ^49^ ^]^ Reads mapped to the mitochondrial genome and to the ENCODE Exclusion List Regions (ENCFF001TDO)^[^ ^50^ ^]^ were removed. Duplicate reads were identified by samtools markdup and removed with samtools view^[^ ^51^ ^]^ prior to peak calling with MACS2 (–nomodel –extsize 150 –shift ‐75 –gsize hs –keep‐dup all).^[^ ^52^ ^]^ ATAC‐seq peaks were intersected with +/− 4 kb of promoter annotations with bedtools intersect.^[^ ^53^ ^]^ Counts in promoter peaks were aggregated with bedtools multicov, followed by library size and peak width normalization.

### Animal Experiments

All animal experiments were performed in accordance with approval (RA/3/100/1336) from the Animal Ethics Committee of the University of Western Australia, Perth. A ZsGreen‐luciferase lentiviral construct was used to create the MDA‐MB‐231‐luc stable cell line. MDA‐MB‐231‐luc was transduced with pLV‐dCas9‐KRAB. Conditions for animal experimentation include: MDA‐MB‐231‐luc wild type, and transfected cells All gRNA and No gRNA. Two million cells were resuspended in 100 µL of serum‐free media and BD Matrigel Matrix High Concentration (BD Bioscience) in a 1:1 ratio. Suspensions were injected subcutaneously into the flanks of 5 week old BALB/c nude females (Animal Resources Centre, WA Australia) with 15 mice per group. Injections and study groups were blind to ensure unbiased results. Bioluminescence analysis was performed on days 4, 7, 11, 14, 18, 21, 25, 28, and 32 post‐inoculation. Animals were monitored for tumor size using a tumor caliper and volumes calculated using the formula: V = Width^2^ × ½ Length. Animals were humanely sacrificed at day 32, day 43, and day 55 and/or when the tumors reached 800 mm^3^ (ethical endpoint).

### Immunofluorescence Staining of Tissue Sections

Paraffin tissue slices were deparaffinized in 3 × 5 min washes of xylene and rehydrated in 2 × 10 min washes of 100% ethanol and 95% ethanol. Tissue slices were subjected to antigen retrieval using heated Citrate buffer (10 mm Sodium Citrate, pH 6) for 10 min, permeabilized in permeabilization buffer (0.2% Triton X‐100 in TBST) for 10 min then washed 2 × 5 min with TBST. Slides were rinsed with 1× PBS and incubated with Sudan black solution (Sigma) for 30 min. Following 3× dipping in 70% ethanol, slides were rinsed 4× in 1× PBS and blocked in 10% normal goat serum with 1% bovine serum albumin (BSA) for 1.5 h at RT. Subsequently, primary antibody was added and incubated overnight in a humidified chamber at 4 °C. The next day, slides were rinsed twice for 5 min in 1× PBS and stained with secondary antibodies Alexa Fluor 488 goat anti‐mouse (1:500, ThermoFisher), Alexa Fluor 594 goat anti‐rabbit secondary antibody (1:500, ThermoFisher), and Hoechst (1:5000, Sigma‐Aldrich). Following a 2 h incubation at RT, slides were mounted with antifade mountant (ThermoFisher) before being sealed. Images were captured using a fluorescent tissue microscope (Nikon), and processed using NIS‐Elements Software.

### Immunohistochemical Staining of Tissue Sections

Similar to IF staining of tissue sections, deparaffinization/rehydration and antigen retrieval steps were carried out. Following incubation in the citrate buffer, sections were washed twice in distilled water for 5 min each before peroxidase blocking for 10 min at RT in 3% hydrogen peroxide. Secondary antibody staining was performed with the EnVision+ Dual Link System‐HRP (DAKO) and signal detection by the Liquid DAB substrate chromagen system (DAKO) as per manufacturer's instructions. To view cellular and tissue structure details, slides were also stained with Mayers Hematoxylin solution (Sigma). Sections were dehydrated with 2× washes with 100% ethanol for 10 s each, followed by 2× washes with xylene for 10 s each. Slides were dried and mounted with DPX Mountant (Sigma). Images were captured using a bright‐field microscope (Nikon), and processed using NIS‐Elements Software.

### Native Chromatin Immunoprecipitation to Assess Chances in Histones Marks

Changes in histone modification were assessed using a modified Native ChIP protocol adapted from Grzybowski et al., 2019^[^ ^54^ ^]^ with minor changes from the original protocol as follows. Native ChIPs were performed in duplicates using Anti‐Rabbit IgG isotype (negative control, CST #66362), Anti‐H3K4me3 (Abcam ab8580), and Anti‐H3K9me3 (Abcam ab8898) in SUM159 All gRNA and No gRNA. Per 6 ChIP reactions, 6 × 10^6^ cells were harvested for nuclei preparation. The M220 Focused‐ultrasonicator (Covaris) was used to sonicate the chromatin for 3 min at 20% duty cycle, 75 W, 200 cycles per burst before proceeding to MNase digestion and HAP purification as per original protocol. 15 µL of HAP purified chromatin was set aside as input control. Following incubation in a C1000 Touch Thermal Cycler (Bio‐Rad) at 75 °C with a hot lid ≥85 °C for 6 min, final clean‐up was performed using 1.5× AMPure beads and purified fragment size and quality were determined using High Sensitivity dsDNA Qubit and Agilent Tapestation. DNA from ChIP input and pulldown were subjected to ThruPLEX DNA‐Seq (Takara R400675) library preparation as per manufacturer's instructions. Libraries were sequenced on the Illumina NovaSeq SP 2 × 61 cycles. Chromatin Immunoprecipitation (ChIP‐seq) data were adapter and quality trimmed with fastp^[^ ^47^ ^]^ with the standard settings followed by mapping with bowtie2^[^ ^48^ ^]^ against the human reference genome hg38 with gnu‐parallel.^[^ ^49^ ^]^ Reads mapped to the ENCODE Exclusion List Regions (ENCFF001TDO)^[^ ^50^ ^]^ were removed. Duplicate reads were identified by samtools markdup and removed with samtools view^[^ ^51^ ^]^ prior to peak calling with MACS2 (–nomodel –extsize 200 ‐75 –gsize hs).^[^ ^52^ ^]^ ChIP‐seq browser tracks were normalized to counts per million (CPM) through deeptools bamCompare (–binSize 10 –normalizeUsing CPM –operation subtract [ChIP – input])^[^ ^55^ ^]^ and saved in BigWig file format.

### Statistical Analysis

In RT‐qPCR experiments, mRNA abundance was normalized to No gRNA control. Percent quantification of colony formation, migration and average length of cells were normalized to wild type. Unless otherwise noted, each experiment was performed on at least three biological replicates and statistical analyses were performed using Prism 9 (GraphPad Software Inc.) software. For in vivo tumorigenicity of ZEB1 repression, *n = 15* mice per test group was determined as statistically significant for bioluminescence imaging. At cull points, *n = 3* mice were sacrificed per group on day 32, day 43, and day 55 for analysis. Statistical analyses were performed using Prism 9 (GraphPad) software with standard Kruskal–Wallis one‐way analysis of variance (one‐way ANOVA) for treatment against the corresponding control, with multiple comparisons. For all tests, differences were considered non‐significant if *p*‐value > 0.05 (ns) and significant at *p* ≤ 0.05 (*), *p* ≤ 0.01 (**), *p* ≤ 0.001 (***), and *p* ≤ 0.0001 (****). Data plotted displays mean ± S.E.M. For RNA‐seq and DNAme analyses the statistical testing was performed using voom‐limma^[^ ^37^ ^]^ with a Benjamini–Hochberg multiple hypothesis correction for adjusted *p*‐values. ChIP‐seq was performed on two replicates of EV and All gRNA whereas ATAC‐seq was performed on one replicate. ChIP‐seq analysis was performed with macs2^[^ ^52^ ^]^ and q‐values from broad peak bed file outputs were used for filtering statistical analysis. macs2 q‐values were calculated from *p*‐values using the Benjamini–Hochberg procedure.

## Conflict of Interest

The authors declare no conflict of interest.

## Author Contributions

Conceptualization: P.B. Methodology: P.B., C.W., and J.C. Formal Analysis: C.W., J.C., and P.B. RNA‐seq Analysis: C.B., J.C., and M.F. DNA‐me Analysis: C.B., M.F., and R.M. ATAC/ChIP‐seq: C.B. and C.P., Investigation: P.B., C.W., E.W. (Edina Wang), E.W. (Eleanor Woodward), A.S., C.W. (Christopher Wallis), C.M., I.G., L.F., A.R., and L.M. Writing Original draft: C.W. and J.C. Review and Editing: P.B., M.F., E.W.T., C.C., M.D., A.T.P., A.R., R.L., and M.E. Figure collation and editing: Figure 1: C.W., J.C., and I.G. Figure 2: C.W. Figure 3: C.W., E.W. (Edina Wang), and A.S. Figure 4: C.W. and E.W. Figure 5: J.C. Figure 6: M.F., C.P., and C.W. Figure 7: R.M. and C.W. Figure 8: C.W. FigureS1, Supporting Information: C.W. FigureS2, Supporting Information: J.C and C.W. FigureS3, Supporting Information: C.W. FigureS4, Supporting Information: C.W. and E.W. (Eleanor Woodward). FigureS5, Supporting Information: C.W., E.W. (Edina Wang), A.S. FigureS6, Supporting Information: E.W. (Eleanor Woodward) and C.W. FigureS7, Supporting Information: C.W., and A.R. FigureS8, Supporting Information: C.W., J.C., and C.W. (Christopher Wallis). FigureS9, Supporting Information: M.F., and C.W. FigureS10, Supporting Information: C.W., and C.P. FigureS11, Supporting Information: R.M. and C.W. FigureS12, Supporting Information: C.W. and E.W. FigureS13, Supporting Information: C.W. and M.F. TableS1, Supporting Information: C.M., C.W., and L.F. TableS2, Supporting Information: A.R. and C.W. TableS3, Supporting Information: C.P. and C.W. Supervision: P.B. Funding Acquisition for this projection: P.B. Project administration: P.B.

## Supporting information

Supporting Information

Supporting Information

## Data Availability

The data discussed in this publication have been deposited in NCBI's Gene Expression Omnibus database and are accessible through GEO Series accession number GSE210277 (https://www.ncbi.nlm.nih.gov/geo/query/acc.cgi?acc=GSE210277).
