# Supplementary material for: Synthetic Epigenetic Reprogramming of Mesenchymal to Epithelial States Using the CRISPR/dCas9 Platform in Triple Negative Breast Cancer
Source: Adv Sci (Weinh). 2023 May 22;10(22):2301802. doi: 10.1002/advs.202301802 (PMC10401103; doi:10.1002/advs.202301802)
Supplement: Supplementary file 1 — Supporting Information [file ADVS-10-2301802-s002.pdf]

ADVANCED SCIENCE
Open Access

Supporting Information

for *Adv. Sci.*, DOI 10.1002/advs.202301802

Synthetic Epigenetic Reprogramming of Mesenchymal to Epithelial States Using the CRISPR/dCas9 Platform in Triple Negative Breast Cancer

*Charlene Waryah, Joseph Cursons, Momeneh Foroutan, Christian Pflueger, Edina Wang, Ramyar Molania, Eleanor Woodward, Anabel Sorolla, Christopher Wallis, Colette Moses, Irina Glas, Leandro Magalhães, Erik W. Thompson, Liam G. Fearnley, Christine L. Chaffer, Melissa Davis, Anthony T. Papenfuss, Andrew Redfern, Ryan Lister, Manel Esteller and Pilar Blancafort**

Supplementary Figure 1.

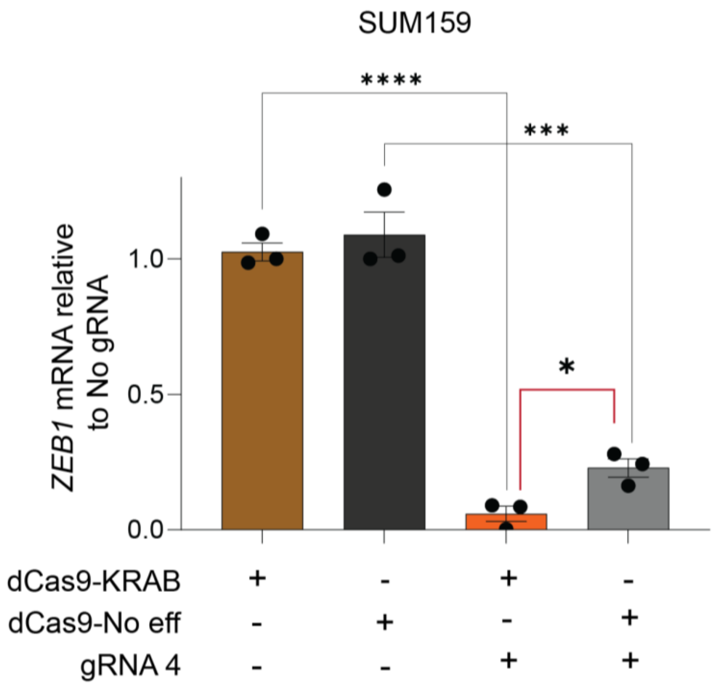

Supplementary Figure 1. Relating to Figure 1: Robust silencing of *ZEB1* requires activity of the KRAB effector domain and the expression of targeting gRNA. Differences in *ZEB1* mRNA transcript abundance in SUM159 cells with various silencing constructs as measured by qRT-PCR (data normalized to No gRNA). Constructs include: dCas9-KRAB in absence of gRNA 4, dCas9 in absence of the effector domain (dCas9-No eff) in absence of gRNA 4, dCas9-KRAB co-expressed with gRNA 4, and dCas9-No eff co-expressed with gRNA 4. *: $p \leq 0.02$, ***: $p \leq 0.001$; ****: $p \leq 0.0001$. Error bars represent S.E.M.

Supplementary Figure 2.

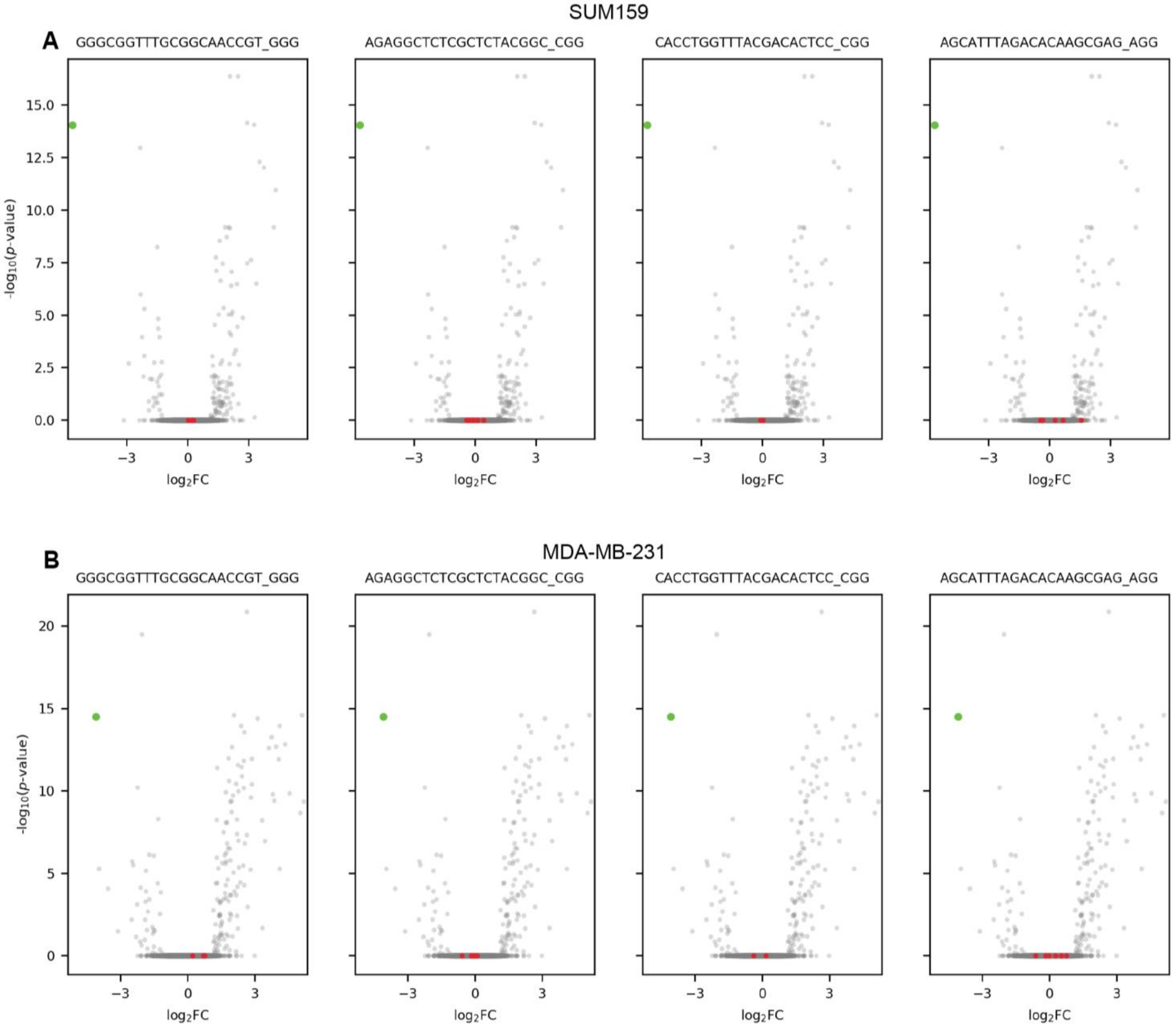

Supplementary Figure 2. Relating to Supplementary Table 1 and Figure 5: Lack of off target activities by dCas9-KRAB gRNA platform in TNBC cell lines. Computationally predicted gRNA off-targets failed to demonstrate significant changes in transcript abundance in the corresponding genes. For our *ZEB1*-targeting gRNAs (*sub-plot titles*) the top predicted off-target sites were identified using Azimuth and Elevation, and mapped to corresponding genes (*red scatter markers*) for the (**A**) SUM159 and (**B**) MDA-MB-231 All gRNA vs No gRNA control RNA-seq data. *ZEB1* is displayed as the green scatter marker. Log_2FC: log2 fold-change.

Supplementary Figure 3.

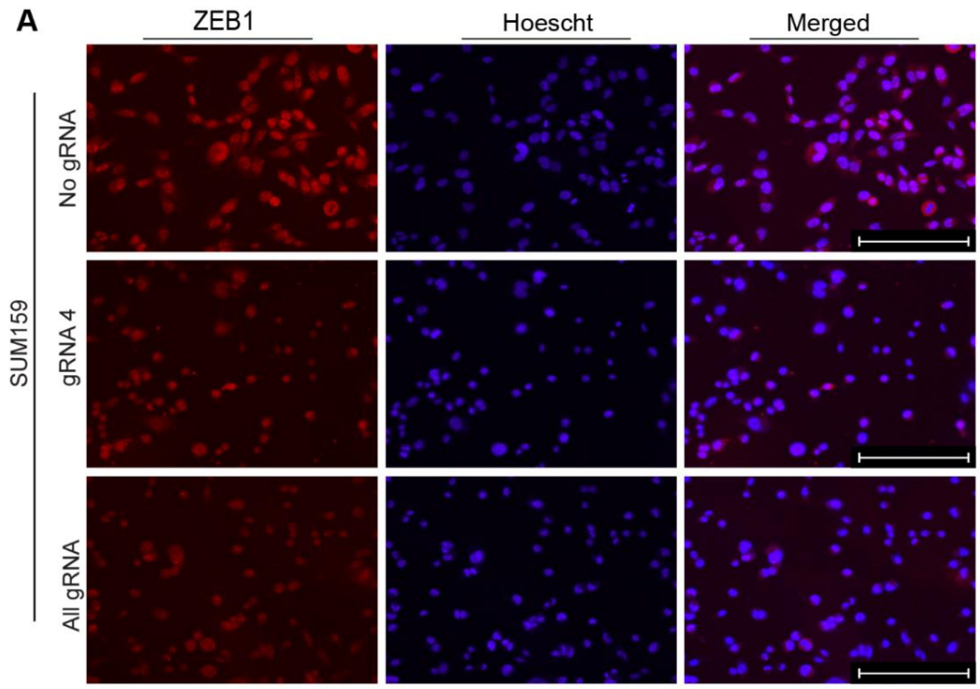

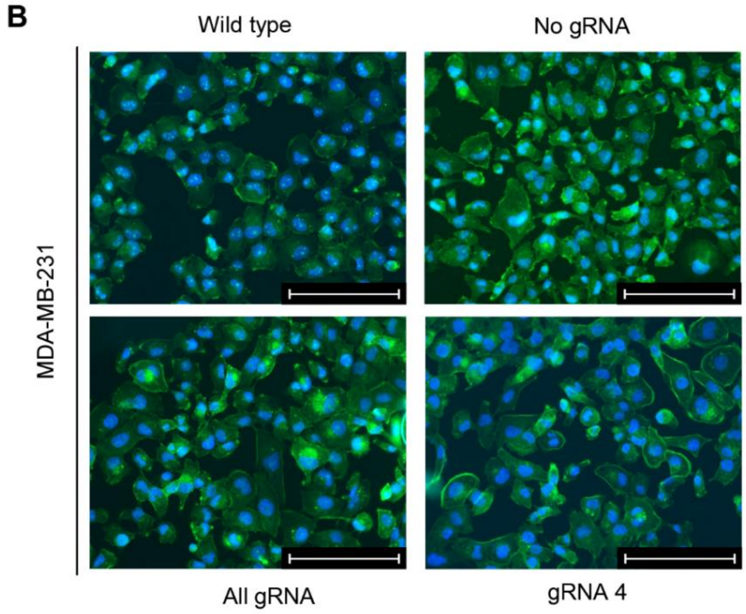

Supplementary Figure 3. Relating to Figure 1D and 2A: Reduced nuclear ZEB1 staining in SUM159 cells with targeting gRNA, and limited morphological changes in treated MDA-MB-231 cells. (**A**) Representative immunofluorescence images of SUM159 No gRNA, gRNA 4 and All gRNA dCas9-KRAB-transduced cells for ZEB1 (*red*) and Hoechst-stained cell nuclei (*blue*). (**B**) Representative immunofluorescence phalloidin stain (*green*) and Hoechst-stained cell nuclei (*blue*) in MDA-MB-231 untransduced, No gRNA, gRNA 4 and All gRNA transduced cells.

Supplementary Figure 4.

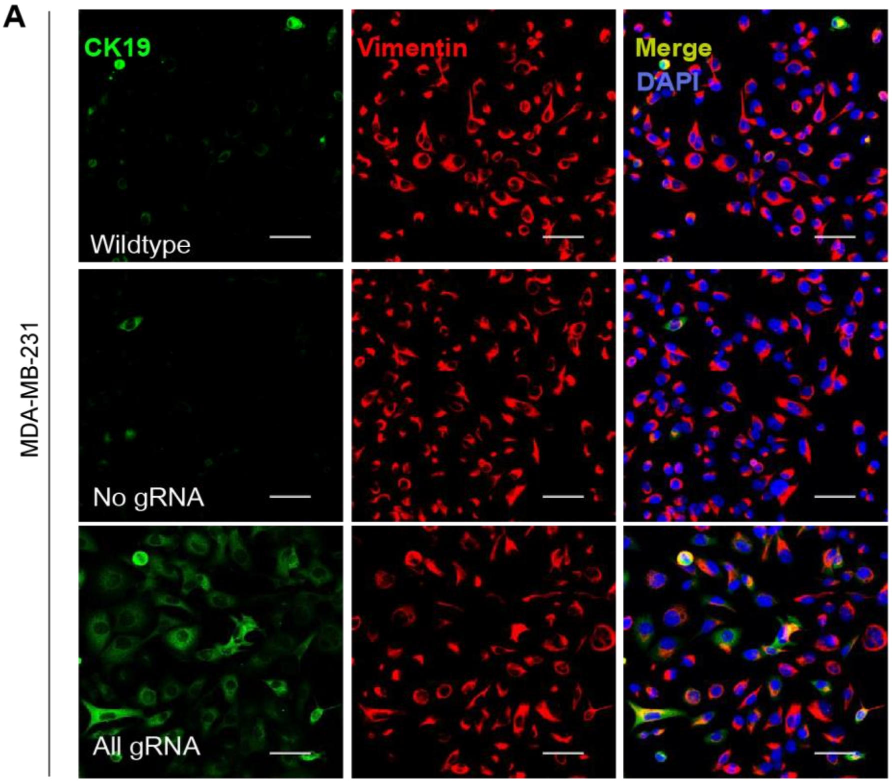

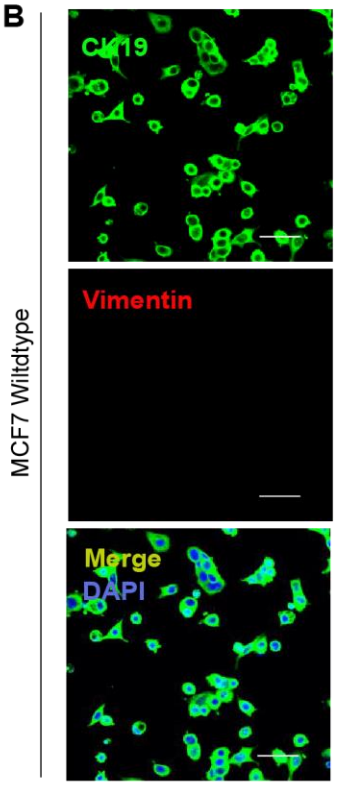

C

Treatment	% Vimentin	%CK19	Expression	CK19 only
Wildtype	97.9 ± 2.9	7.1 ± 0.8	7.1 ± 0.8	0.0 ± 0.0
No gRNA	98.6 ± 1.3	5.5 ± 6.2	5.5 ± 6.2	0.0 ± 0.0
All gRNA	88.3 ± 6.3	28.3 ± 1.6	22.2 ± 3.2	6.1 ± 2.9

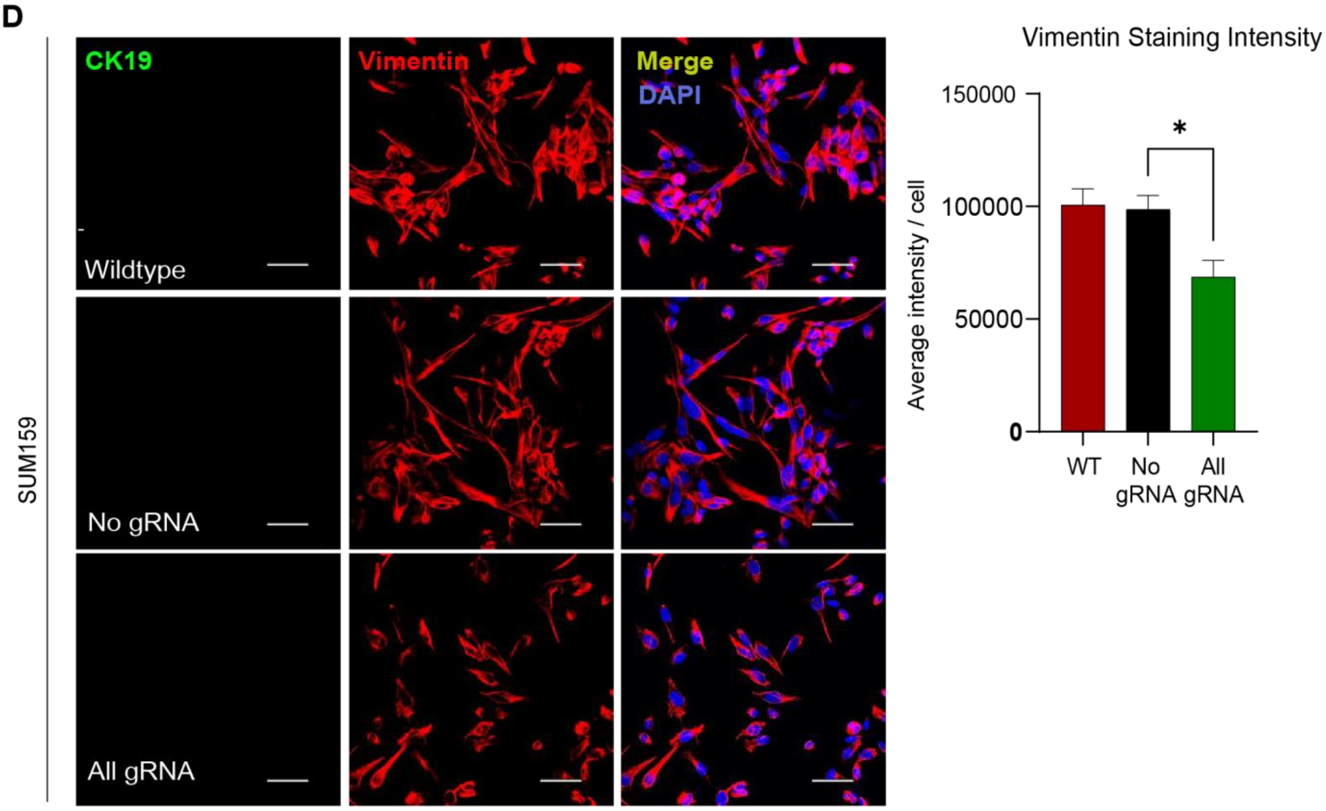

Supplementary Figure 4. Relating to Figure 1D and Supplementary Figure 3: Visualization of CK19 and Vimentin in SUM159 and MDA-MB-231 cell lines. (**A**) Immunofluorescence for the visualization of CK19 (*green*) and Vimentin (*red*) in No gRNA and All gRNA dCas-KRAB transduced MDA-MB-231. (**B**) MCF7 cell line wildtype control was included as a positive control for CK19 and negative control for Vimentin. (**C**) Table detailing the percent positive cells containing Vimentin and/or CK19 in the MDA-MB-231 cell lines. (**D**) Immunofluorescence for the visualization of CK19 (*green*) and Vimentin (*red*) in No gRNA and All gRNA dCas-KRAB transduced SUM159. Staining intensity of Vimentin in the SUM159 cells provided *at right*. DAPI stain (*blue*) is indicated to label the nuclei. CK19: cytokeratin 19, WT: wildtype.

Supplementary Figure 5.

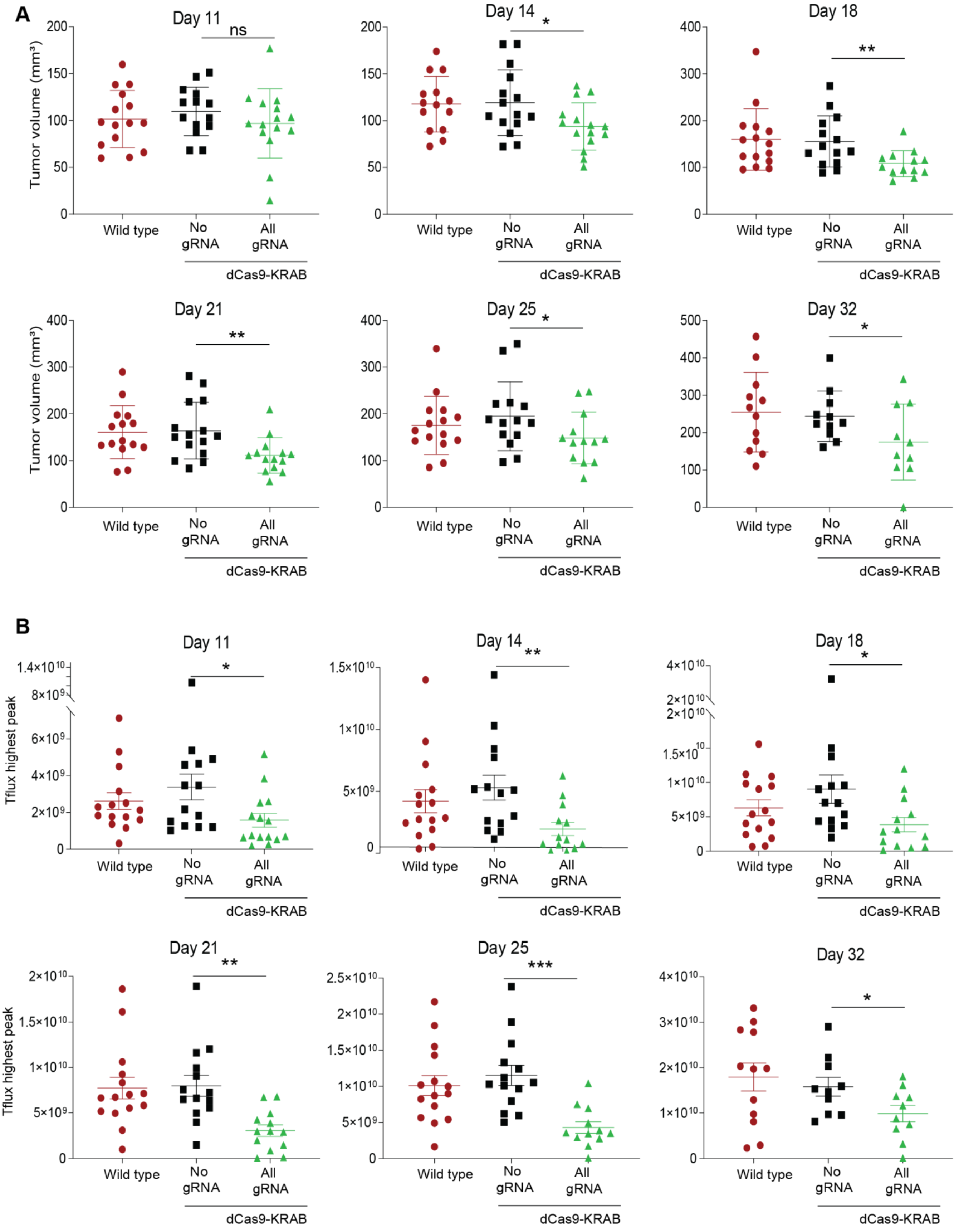

Supplementary Figure 5. Relating to Figure 3: Repression of ZEB1 *in vivo* suppresses the growth of xenograft tumor models. (**A**) Tumor growth inhibition *in vivo* of MDA-MB-231-luc xenografts at days 11, 14, 18, 21, 25 and 32 post-implantation of the cells. Each dot represents one mouse. Significance was calculated using Graphpad Prism with ns = non-significant, *p ≤0.05, **p ≤0.01 and ***p ≤0.001. Error bars represent S.E.M. (**B**) Tflux peaks obtained from bioluminescence imaging at days 11, 14, 18, 21, 25 and 32 post-inoculation of the cells. Each dot represents one individual mouse. Significance was calculated using GraphPad Prism with ns = non-significant, *p ≤0.05, **p ≤0.01 and ***p ≤0.001. Error bars represent S.E.M.

Supplementary Figure 6.

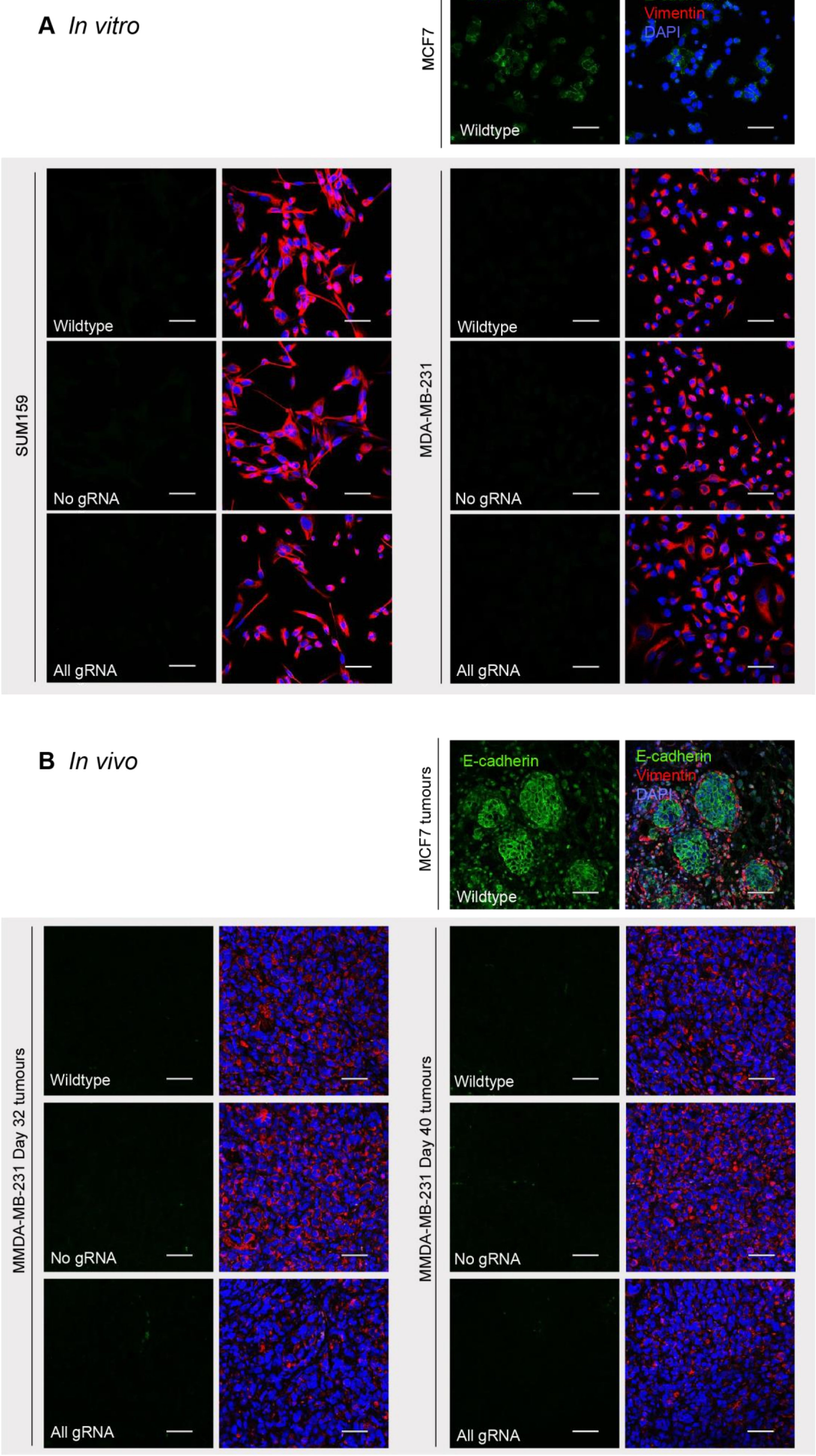

Supplementary Figure 6: **Visualization of E-cadherin and Vimentin in TNBC cell lines and MDA-MB-231 Day 30 and Day 40 tumors.** Immunofluorescence for the visualization of E-cadherin (*green*) and Vimentin (*red*) in No gRNA and All gRNA dCas-KRAB transduced SUM159 and MDA-MB-231 cell lines (**A**). DAPI stain (*blue*) is indicated to label the nuclei. MCF7 cell line wildtype control was included as a positive control for E-cadherin and negative control for Vimentin. (**B**) MDA-MB-231 wildtype, No gRNA and All gRNA tumors stained at Day 32 and Day 40 for the visualization of E-cadherin (*green*) and Vimentin (*red*). MCF7 tumor was stained as a positive control for E-cadherin and negative control for Vimentin.

Supplementary Figure 7.

A Histological examples of CK19:Vim co-stained mouse xenografts

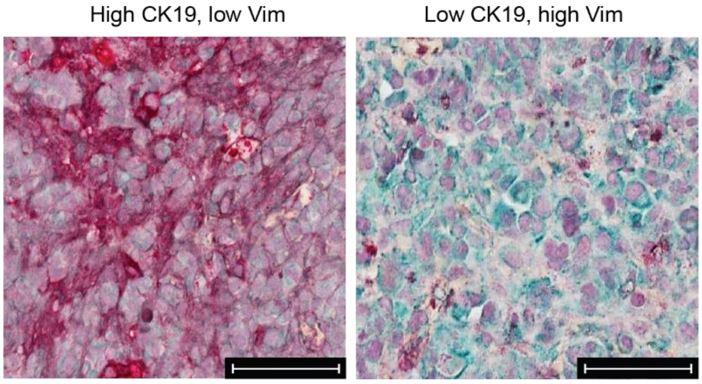

B Day 32: expression of pro-mesenchymal transcription factors

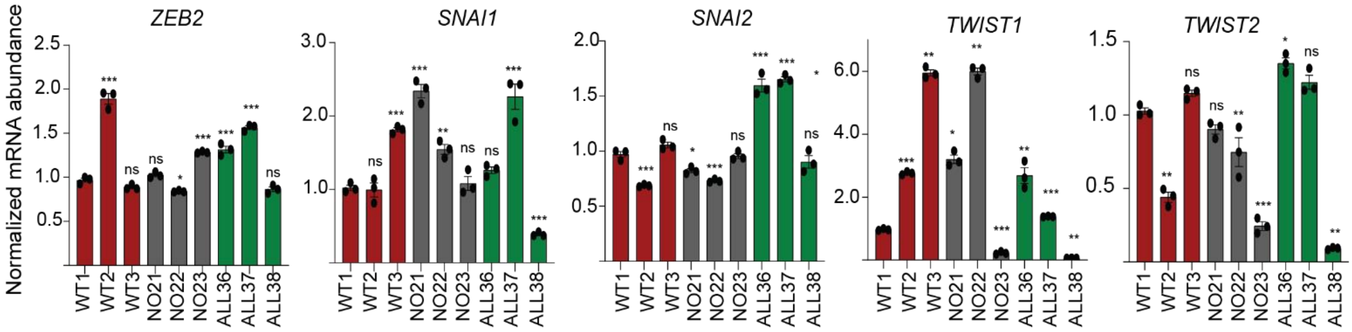

C Day 43: expression of pro-mesenchymal transcription factors

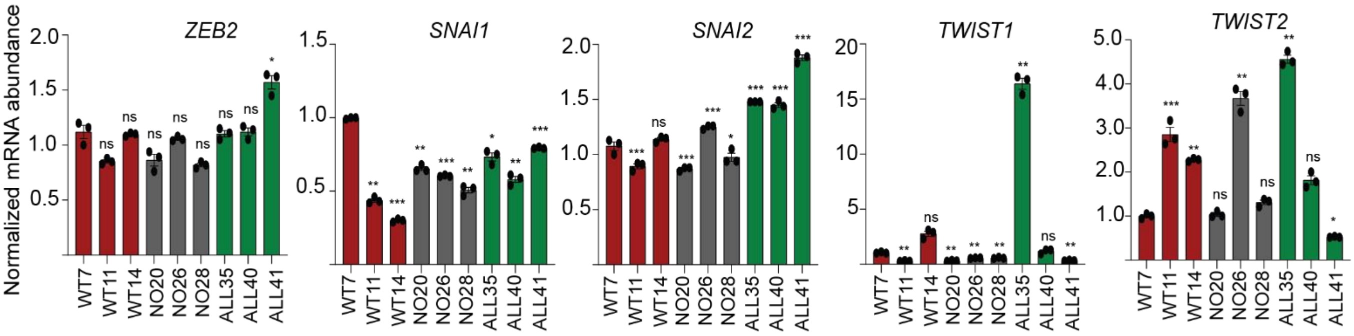

D Day 55: expression of pro-mesenchymal transcription factors

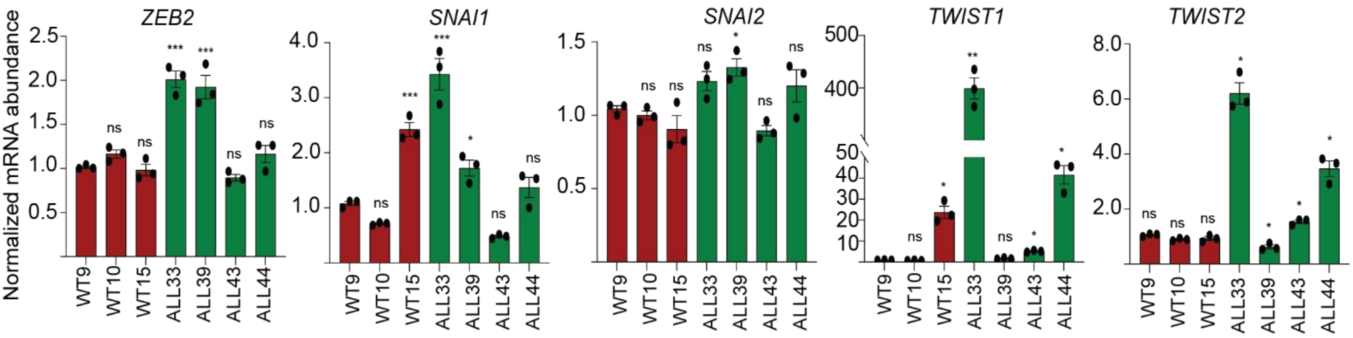

Supplementary Figure 7. Relating to Supplementary Table 2 and Figure 3D: Repression of ZEB1 *in vivo* reactivates the pro-epithelial miRNAs of the 200 family and modulates EMT-TF expression. (**A**) Histological examples of CK19:Vim (*pink:blue*) dual stain used in mouse xenografts demonstrating areas of predominantly CK19 staining (*left*) and predominantly Vim (*right*) staining. Gene expression analysis by qRT-PCR to determine mRNA levels of EMT-transcription factors *ZEB2*, *SNAI1*, *SNAI2*, *TWIST1* and *TWIST2* in tumors harvested at: (**B**) day 32, (**C**) day 43 and (**D**) day 55. Significance was calculated using Prism with **p* ≤0.05, ***p* ≤0.01, ****p* ≤0.001, ns = non-significant. Error bars represent S.E.M. WT: Wild type, NO: No gRNA, ALL: All gRNA.

Supplementary Figure 8.

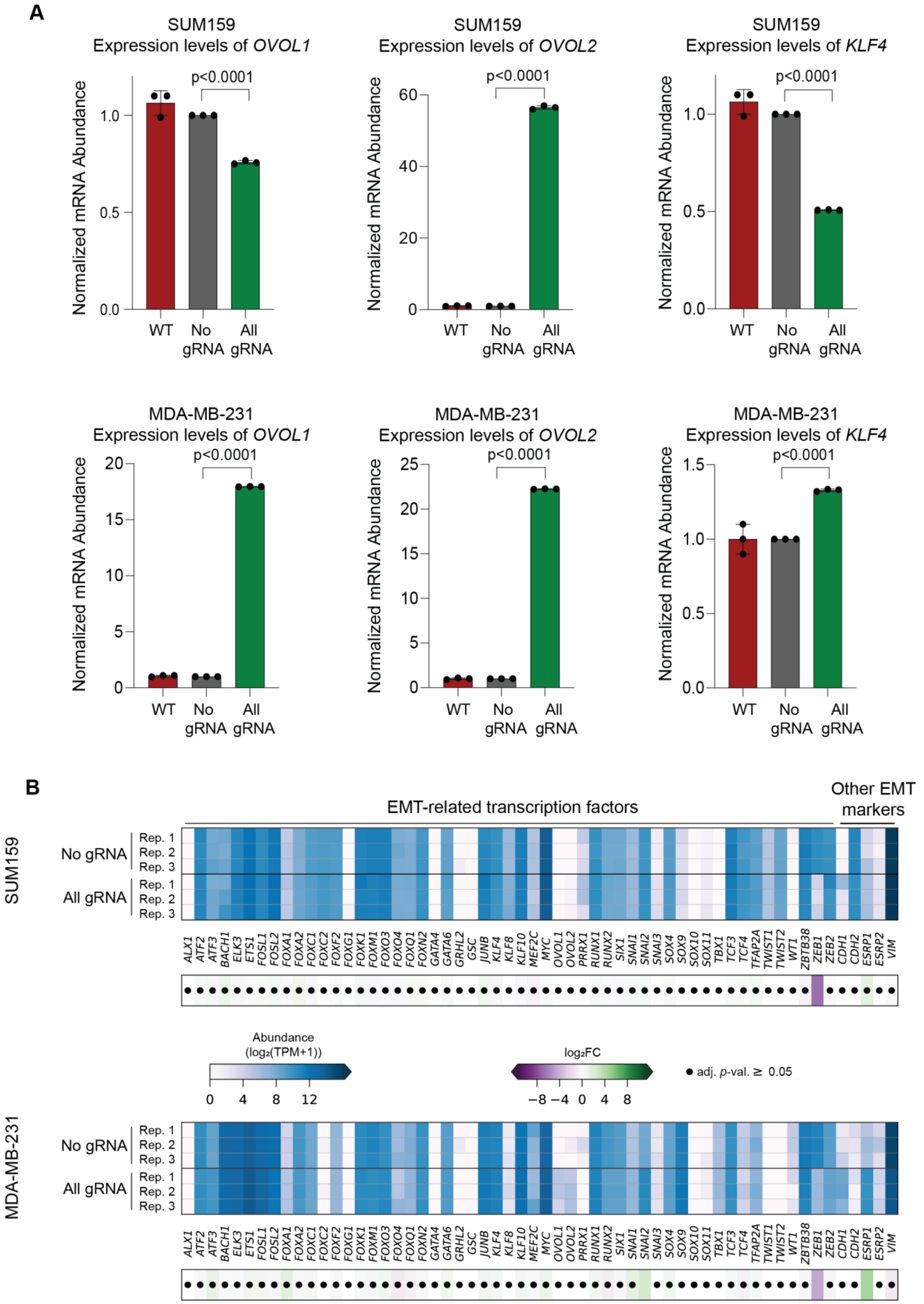

Supplementary Figure 8: Changes in the transcript abundance of EMT-related transcription factors. (**A**) The normalized abundance of *OVOL1*, *OVOL2* and *KLF4* RNA transcripts in SUM159 cells (*top row*) and MDA MB 231 cells (*bottom row*) under different treatment conditions. Significance was calculated using Prism and error bars represent S.E.M. (**B**) The abundance (log|TPM+1|) and log fold-change (logFC) for a selection of EMT-related transcription factors and EMT markers within SUM159 and MDA-MB-231 cells in the presence and absence of ZEB1-targeting gRNAs for the dCas9-KRAB system.WT: WIldtype, Rep: replicate, adj. *p*-val.: adjusted *p*-value

Supplementary Figure 9.

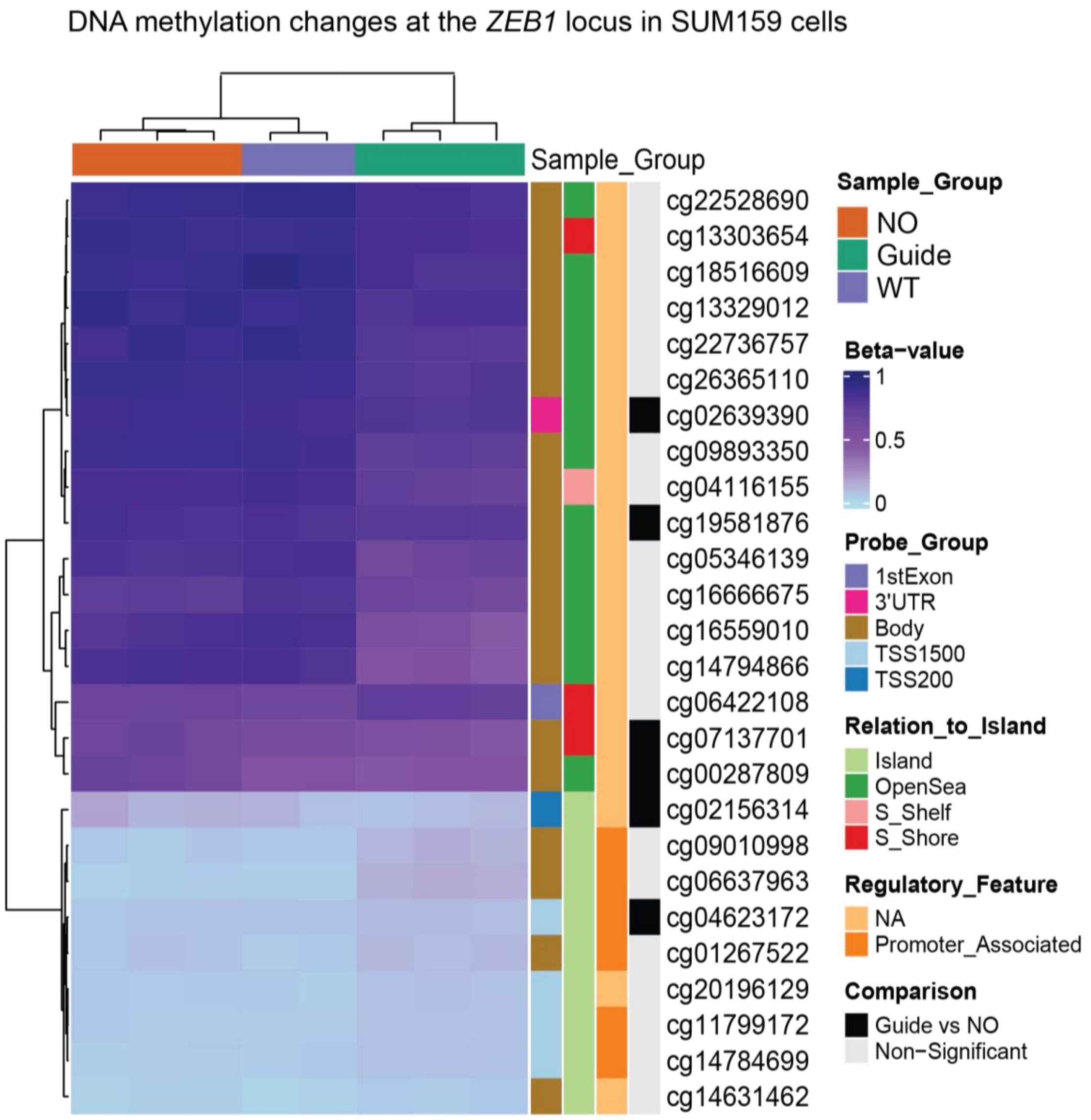

Supplementary Figure 9. Relating to Figure 6A: DNAme changes in the *ZEB1* locus following dCas9 silencing, with increased DNAme in promoter-associated and several gene body probes, but decreased DNAme in numerous gene body probes. β-values for differentially-methylated probes associated with *ZEB1* (*at left*) in the wild type (WT), No gRNA (NO) and All gRNA (Guide) conditions for SUM159 cells (*annotated at top*). Probes were selected if they were differentially expressed for All gRNA vs WT and/or All-gRNA vs NO (*Comparison; annotated at right*). Probes are also annotated with the probe group (position relative to the gene; *Probe_Group*), position relative to the nearby-CpG islands (*Relation_to_Island*) and associations with the ZEB1 promoter (*Regulatory_Feature*). TSS: transcriptional start site, 3'UTR: three prime untranslated region.

Supplementary Figure 10.

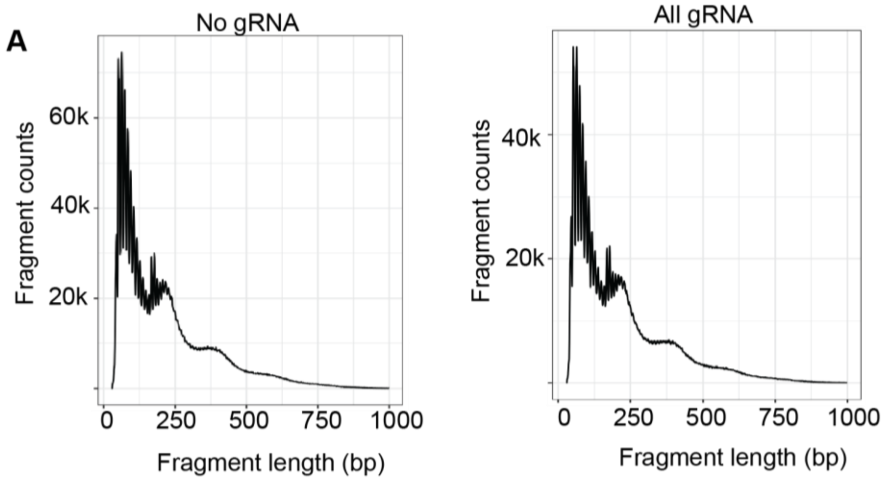

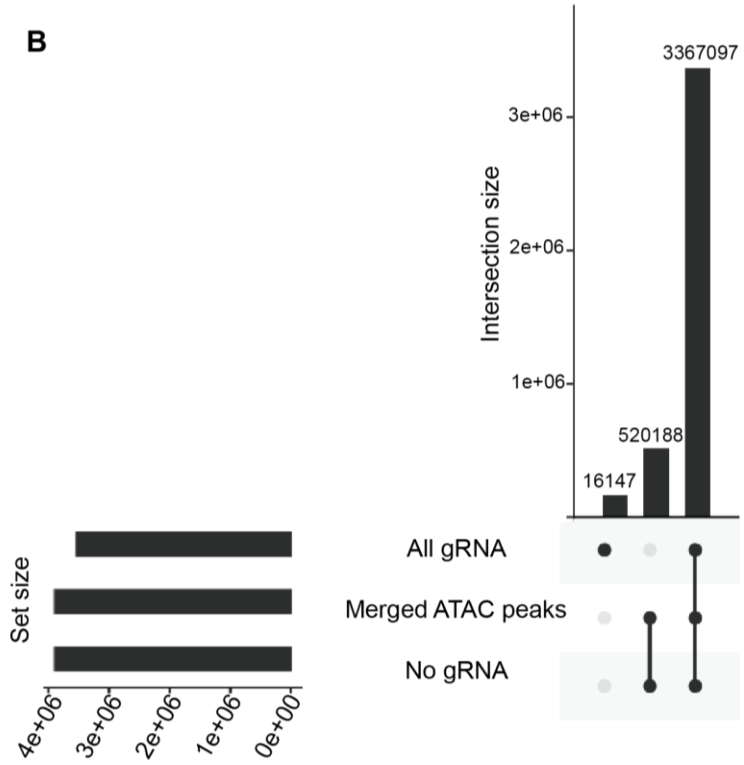

Supplementary Figure 10. Relating to Figure 6D: ATAC generated libraries pass stringent post-alignment processing quality control. (**A**) Post alignment processing of assay of transposase accessible chromatin (ATAC) sequencing data with quality control performed to assess insert sizes for libraries with fragment counts and fragment length (bp). (**B**) Peak calling performed to identify areas enriched with aligned reads. Overlapping peaks between the groups were visualized using an UpSet plot. Bp: base-pair.

Supplementary Figure 11.

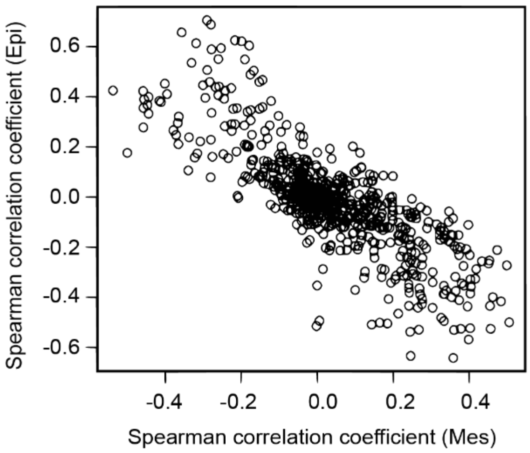

Supplementary Figure 11. Relating to Figure 7: Differentially methylated probes from our SUM159 model show a strong inverse association with epithelial score and mesenchymal score across TCGA tumor samples. A scatter plot showing the Spearman's correlation between probe β-value and corresponding bulk tumor mesenchymal score (determined by gene set scoring of RNA-seq data; *x-axis*) and epithelial score (*y-axis*) for probes that were differentially methylated in SUM159 cells with ZEB1 repression, from genes that were differentially expressed in SUM159 cells with ZEB1 repression (All gRNA vs No gRNA control). Note that only probes that are consistent between the 850k (*data from this study*) and 450k (*used for TCGA BRCA data*) Beadarray/DNAme platforms could be included. TCGA-BRCA: The Cancer Genome Atlas Breast Invasive Carcinoma, Epi: epithelial, Mes: mesenchymal.

Supplementary Figure 12.

A *In vitro*

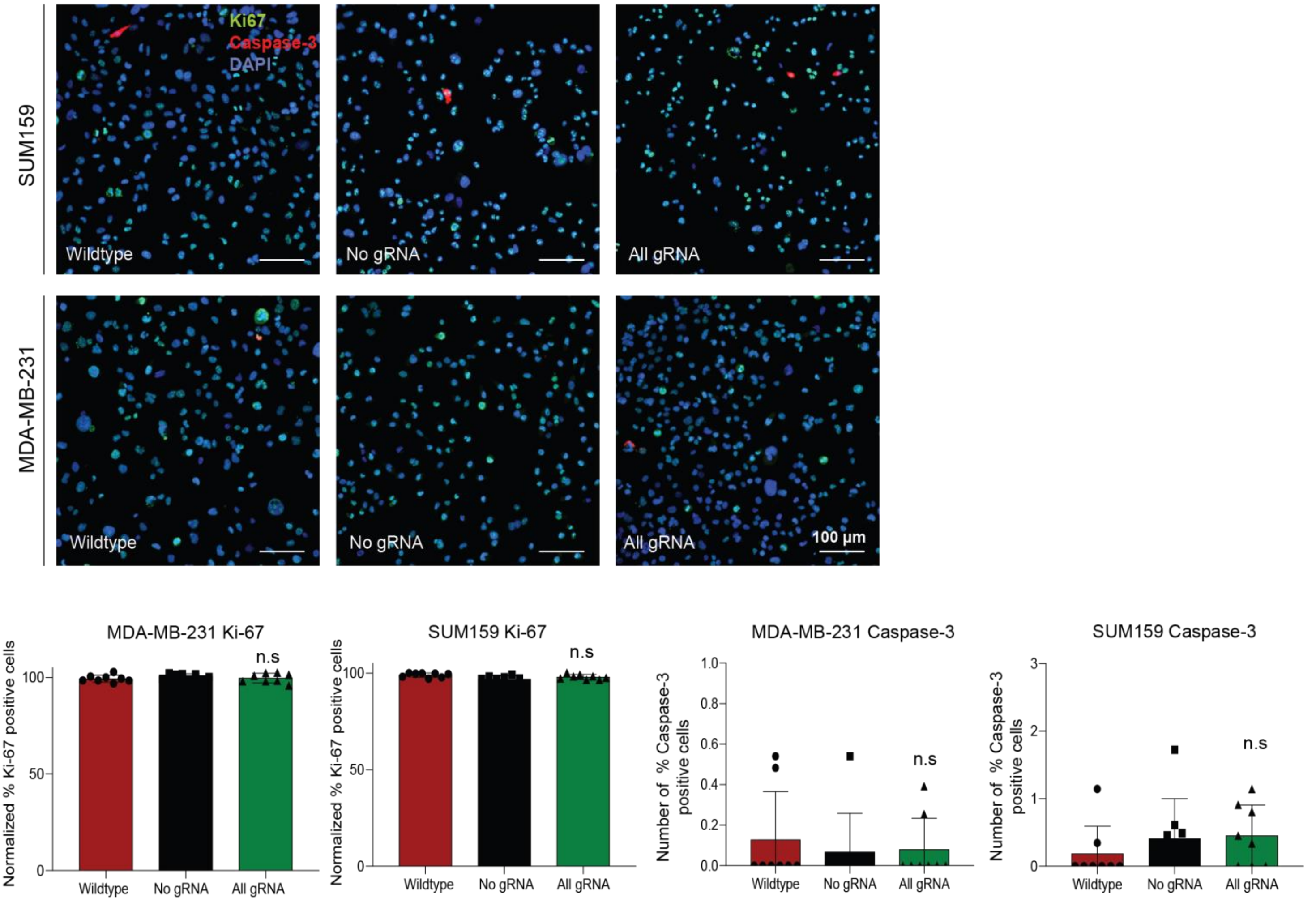

B *In vivo*

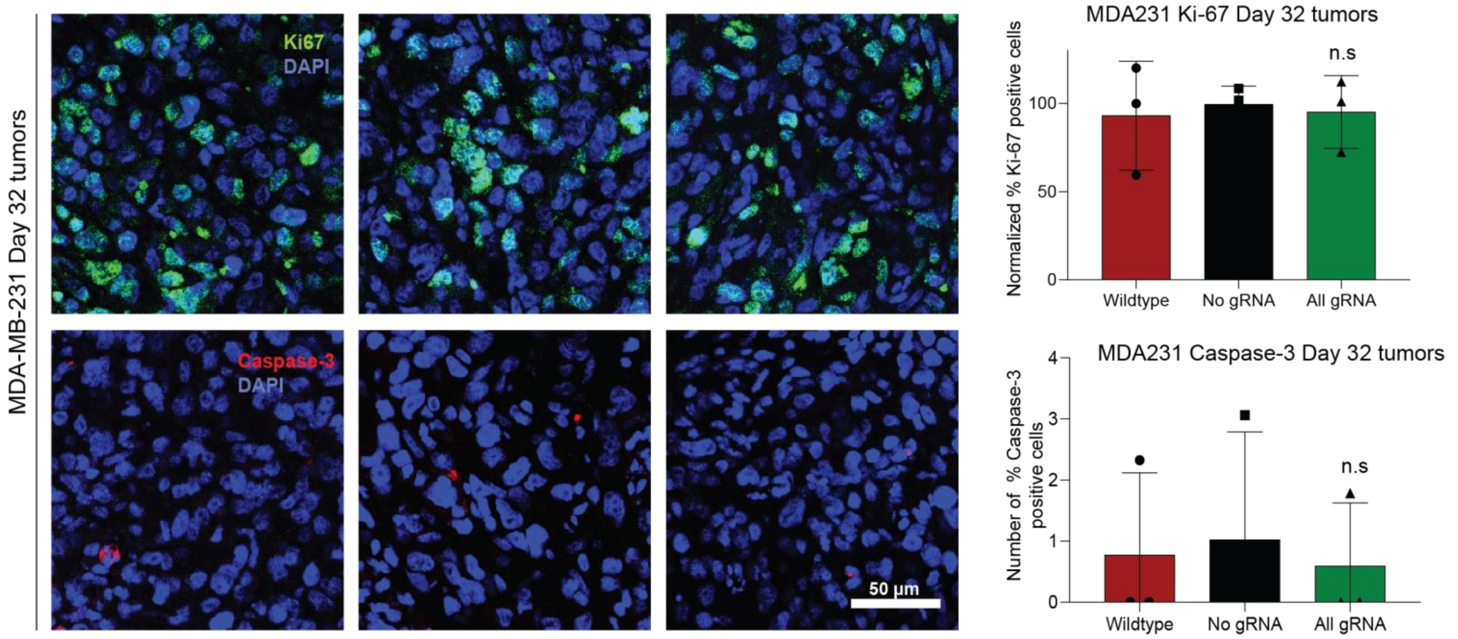

Supplementary Figure 12: Assessment of Ki-67 and Caspase-3 in TNBC cell lines and MDA-MB-231 Day 30 tumors. Representative immunofluorescence images for the visualization of Ki-67 (*green*) and Caspase-3 (*red*) in No gRNA and All gRNA dCas-KRAB transduced SUM159 and MDA-MB-231 cell lines (**A**). DAPI stain (*blue*) is indicated to label the nuclei. (**B**) Day 32 tumors of MDA-MB-231 wildtype, No gRNA and All gRNA tumors stained for the visualization of Ki-67 (*green*) and Caspase-3 (*red*). n.s: non-significant

Supplementary Figure 13.

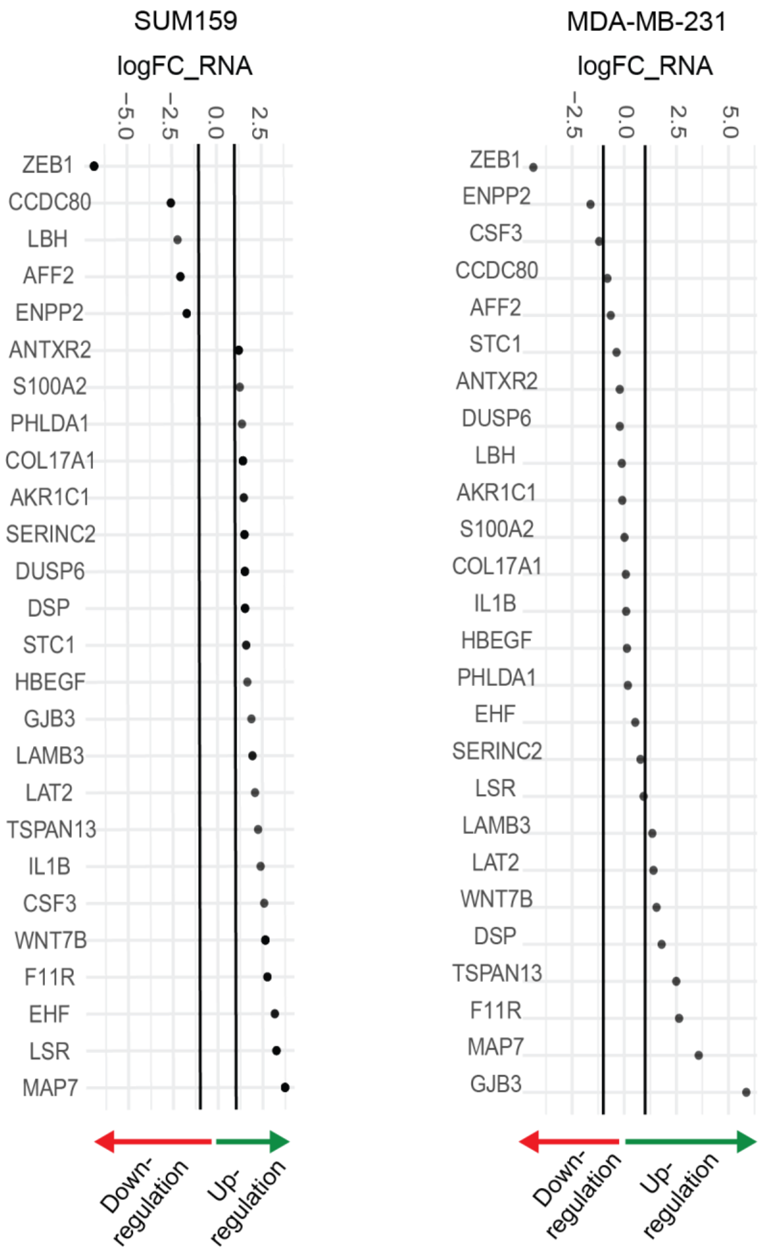

Supplementary Figure 13: Relating to Figure 6 and Figure 7. Changes in the transcript abundance. RNA-sequencing profiling of the 26-gene signature in a side by side comparison of SUM159 and MDA-MB-231. logFC_RNA: Log 2 fold-change.
