# Supplementary material for: Synthetic Epigenetic Reprogramming of Mesenchymal to Epithelial States Using the CRISPR/dCas9 Platform in Triple Negative Breast Cancer
Source: Adv Sci (Weinh). 2023 May 22;10(22):2301802. doi: 10.1002/advs.202301802 (PMC10401103; doi:10.1002/advs.202301802)
Supplement: Supplementary file 2 — Supporting Information [file ADVS-10-2301802-s001.pdf]

ADVANCED SCIENCE
Open Access

Supporting Information

for *Adv. Sci.*, DOI 10.1002/advs.202301802

Synthetic Epigenetic Reprogramming of Mesenchymal to Epithelial States Using the CRISPR/dCas9 Platform in Triple Negative Breast Cancer

*Charlene Waryah, Joseph Cursons, Momeneh Foroutan, Christian Pflueger, Edina Wang, Ramyar Molania, Eleanor Woodward, Anabel Sorolla, Christopher Wallis, Colette Moses, Irina Glas, Leandro Magalhães, Erik W. Thompson, Liam G. Fearnley, Christine L. Chaffer, Melissa Davis, Anthony T. Papenfuss, Andrew Redfern, Ryan Lister, Manel Esteller and Pilar Blancafort**

Supplementary Table 1: sgRNA design and bioinformatics off-target predictions

Supplementary Table 1A: ZEB1 gRNA repression sequences					
Guide	Strand	Sequence	PAM	Specificity Score	Efficiency Score
1	-1	GGGCGGTTTGCGGCAACCGT	GGG	96.5	50.5
2	-1	AGAGGCTCTCGCTCTACGGC	CGG	89.9	58.6
3	-1	CACCTGGTTTACGACACTCC	CGG	88.3	61.3
4	1	AGCATTTAGACACAAGCGAG	AGG	80.7	67.6

Supplementary Table 1B: Top 8 Off-target prediction				
Strand	Potential off target sequence	Mis matches	Gene	Hg38 target sequence
1	ACCGCGTCCCTACGGTTTCC_CGG	3	NIL	ACCGCGGCCCCACGGCTTCC_TGT
1	GCGGATGCCGGGAAACCGTA_GGG	2	MPPED1	GCGGATGCCAGGAGACCGTA_AGC
-1	GCGGATGCCGGGAAACCGTA_GGG	3	TSSC1	GCGGATGCCAGGAAACCTTG_GTG
1	GCGGATGCCGGGAAACCGTA_GGG	3	NIL	GCGGATGCCAGGAAACCTTG_GTG
-1	GCGGATGCCAGGAAACCTTG_GTG	3	ROBO2	CGGGCTGTGTGCGGCAATCG_CAG
-1	GCGGATGCCAGGAAACCTTG_GTG	3	RABGGTA	CGGGCGGGTAGCGGCAACCT_TTG
-1	GCGGATGCCAGGAAACCTTG_GTG	3	PLD4	CGGGCGGTTTGCGACGCCCG_GGC
1	GCGGATGCCAGGAAACCTTG_GTG	3	GNAS	CGGGCGCTCTGCGGCAAGCG_GTG

gRNA were designed using Benchling (benchling.com). Strand -1: reverse strand gRNA. Strand 1: forward strand guide.
PAM: protospacer-adjacent motif.

Supplementary Table 1. Relating to Supplementary Figure 2 and Figure 5: Predicted gRNA specificity and efficiency scores and top eight off-targets. (**A**) Four ZEB1-targeting gRNAs (labelled G1 – G4) sequence and PAM recognition sites are outlined with high specificity and efficiency scores designed by Benchling. (**B**) Top 8 potential predicted off-target sequences (mismatches <3) were identified using Azimuth and Elevation, and mapped to corresponding Hg38 target sequences. PAM: protospacer adjacent motif, Strand -1: reverse strand gRNA. Strand 1: forward strand guide, Hg38: Genome Research Consortium human build 38.

Supplementary Table 2: Histological examination of CRISPR edited tumours

A) individual tumor assessments

Treatment	Timepoint	CK19		Vimentin				co-expression	% necrosis	size - mm2 width x breadth
		Net intensity	%	Intensity centre	%	Intensity edge	% gain	%		
WT	early	0.75	35	0.5	13	1	20	0	1.8	38%
WT	early	1	5	0.5	18	0.5	18	0	0.3	0%
WT	early	0.25	52	0.5	8	0.5	12	0	4.3	43%
WT	early	0.67	31	0.5	12	0.67	17	0	1.8	27%
WT	late	0.5	31	0.5	12	0.5	14	0	2.2	48%
WT	late	0.5	35	0.5	15	0.5	15	0	2.3	50%
WT	late	0.5	28	0.5	8	0.5	12	0	2.3	45%
WT	late	0.5	31	0.5	12	0.5	14	0	2.2	48%
No gRNA	early	1.2	22	0.5	20	0.75	32	0	0.7	42
No gRNA	early	0.2	2	0.75	38	1	48	0	0.0	0
No gRNA	early	1	20	0.5	20	1	60	2	0.3	40
No gRNA	early	0.8	15	0.6	26	0.9	46	1	0.3	27
No gRNA	late	0.5	5	1.8	80	2	90	1	0.1	58
No gRNA	late	1.2	25	0.5	15	0.8	30	0	0.8	55
No gRNA	late	0.5	18	0.5	38	1.2	58	5	0.3	2
No gRNA	late	0.7	16	0.9	46	0.7	59	2	0.3	38
All gRNA	early	0.5	5	1	35	1	38	0	0.1	2%
All gRNA	early	1	45	0.5	10	0.5	12	0	3.8	60%
All gRNA	early	1	40	0.5	18	0.75	38	5	1.1	42%
All gRNA	early	0.67	30	0.67	21	0.75	29	2	1.0	35%
All gRNA	late	0.5	32	0.5	12	0.75	32	2	1.0	50
All gRNA	late	1	25	0.5	22	0.5	28	8	0.9	62
All gRNA	late	0.5	12	0.5	5	0.5	10	0	1.2	48
All gRNA	late	0.67	23	0.5	13	0.6	23	3	1.0	53

B) summated tumor assessments

Treatment	Timepoint	CK19		Vimentin				co-expression	% necrosis	size - mm2 width x breadth
		Net intensity	%	Intensity centre	%	Intensity edge	% gain	%		
WT	early	0.67	31	0.5	12	0.7	17	0	27	18.5
WT	late	0.5	31	0.5	12	0.5	14	0	48	18.5
No gRNA	early	0.8	15	0.6	26	0.9	46	1	27	19
No gRNA	late	0.7	16	0.9	46	0.7	59	2	38	18
All gRNA	early	0.67	30	0.7	21	0.8	29	2	35	15
All gRNA	late	0.67	33	0.5	13	0.6	23	3	53	21

Supplementary Table 2. Relating to Figure 3 and Figure 4: Histological examination of tumors extracted from xenograph tumor models. (**A**) Staining of CK19 and Vimentin, with net intensity and % of stain, in tumors extracted from MDA-MB-231 wild type, All gRNA and No gRNA xenograft model in nude mice. Tumors extracted were assessed at early and late timepoints representing day 32 and day 43 post-inoculation, respectively. (**B**) Cumulative tumor assessment of each treatment group, timepoint and summated intensity and percentages. WT: untransduced tumors, early timepoint: day 32, late timepoint: day 43. CK19: cytokeratin 19

Supplementary Table 3: ChIP sequencing revealed a number of genes with significant changes in H3K9me3

Gene	Description	Chromosome (start-end) Hg38	Width (bp)	logFC	Adj. *p*-value	Description and relation to EMT	Ref
ZEB1	zinc finger E-box binding homeobox 1	Chr10 31306777-31328378<br>Chr10 31360865-31366033	21602<br>5169	1.421<br>0.689	0.000	EMT known transcriptional repressor	[1]
PMAIP1	phorbol-12-myristate-13-acetate-induced protein 1	Chr18 59904411-59906016	1606	1.437	0.001	Pro-apoptotic gene inhibited by ZEB1	[2]
MANBA	mannosidase beta	Chr4 102717939-102721258	3320	-0.658	0.000	Glycan degradation; glycans play a role in EMT	[3]
TMC2	transmembrane channel like 2	Chr20 2624599-2627755	3157	-0.733	0.006	Transmembrane protein; no link to EMT	[4]
PMS1	PMS1 homolog 1, mismatch repair system component	Chr2 189810654-189812295	1642	-1.369	0.006	Forms a dimer with MLH1, a DNA mismatch repair protein; no link to EMT	[5,6]
LAMA3	laminin subunit alpha 3	Chr18 23720542-23722925	2384	-0.713	0.004	Involved in cell adhesion, attachment and basement membrane organization; Regulated by ZEB1	[7]
PCDHB5	protocadherin beta 5	Chr5 141138879-141140567	1689	0.842	0.013	Potential cell adhesion protein; Likely role as cadherin switching play a fundamental role in EMT-MET	[8]
HS6ST3	heparan sulfate 6-O-sulfotransferase 3	Chr13 96730654-96732534	1881	-0.840	0.021	Involved in tumorigenesis in breast cancer; no link to EMT	[9]
CTNNA3	catenin alpha 3	Chr10 66951042-66952442	1401	0.969	0.038	Modulates cell adhesion by coupling cadherin to actin skeleton; pro-epithelial marker	[10]
LRRTM3	leucine rich repeat transmembrane neuronal 3	Chr10 66951042-66952442	1401	0.969	0.033	Postsynaptic adhesion molecule; May be regulated by pro-epithelial miR-200b	[11]<br>[12]
AOX1	aldehyde oxidase 1	Chr2 200606098-200608010	1913	-0.672	0.033	Cytosolic enzyme, physiological role unknown; no link to EMT	[13]
IHO1	interactor of HORMAD1 1	Chr3 49238345-49239847	1503	-0.825	0.041	Regulation of chromosome segregation; no link to EMT	[14]
TSPAN14	tetraspanin 14	Chr10 80476987-80478909	1923	-0.710	0.039	Interacts with cell surface proteins involved in adhesion; link to EMT	[15]
PUS10	pseudouridine	Chr2	1347	-0.854	0.049	Promotes miRNA	[16]

	synthase 10	61021496-61022842				biosynthesis; No link to EMT	
PEX13	peroxisomal biogenesis factor 13	Chr2 61021496-61022842	1347	-0.854	0.048	Involved in metabolic reactions, oxidation of biomolecules; No link to EMT	17

Supplementary Table 3: Relating to Figure 6D: Genome-wide epigenetic reprogramming by *ZEB1* silencing reports significant changes genes crucial in remodelling cell adhesion. Native ChIP sequencing identified genome-wide changes in H3K9me3 in EMT related genes and the degree of histone modification. Hg38: Genome Research Consortium human build 38, bp: base pair, Adj. *p*-value: adjusted *p*-value, EMT: Epithelial to mesenchymal transition, Ref: reference.

References

1 Dongre, A. & Weinberg, R. A. New insights into the mechanisms of epithelial-mesenchymal transition and implications for cancer. *Nat Rev Mol Cell Biol* **20**, 69-84, doi:10.1038/s41580-018-0080-4 (2019).

2 Sanchez-Tillo, E. *et al.* The EMT activator ZEB1 promotes tumor growth and determines differential response to chemotherapy in mantle cell lymphoma. *Cell Death Differ* **21**, 247-257, doi:10.1038/cdd.2013.123 (2014).

3 Zhang, J., Ten Dijke, P., Wuhrer, M. & Zhang, T. Role of glycosylation in TGF-beta signaling and epithelial-to-mesenchymal transition in cancer. *Protein Cell* **12**, 89-106, doi:10.1007/s13238-020-00741-7 (2021).

4 Pan, B. *et al.* TMC1 Forms the Pore of Mechanosensory Transduction Channels in Vertebrate Inner Ear Hair Cells. *Neuron* **99**, 736-753 e736, doi:10.1016/j.neuron.2018.07.033 (2018).

5 Plys, A. J., Rogacheva, M. V., Greene, E. C. & Alani, E. The unstructured linker arms of Mlh1-Pms1 are important for interactions with DNA during mismatch repair. *J Mol Biol* **422**, 192-203, doi:10.1016/j.jmb.2012.05.030 (2012).

6 Heck, J. A. *et al.* Negative epistasis between natural variants of the Saccharomyces cerevisiae MLH1 and PMS1 genes results in a defect in mismatch repair. *Proc Natl Acad Sci U S A* **103**, 3256-3261, doi:10.1073/pnas.0510998103 (2006).

7 Drake, J. M. *et al.* ZEB1 coordinately regulates laminin-332 and {beta}4 integrin expression altering the invasive phenotype of prostate cancer cells. *J Biol Chem* **285**, 33940-33948, doi:10.1074/jbc.M110.136044 (2010).

8 Andrews, J. L., Kim, A. C. & Hens, J. R. The role and function of cadherins in the mammary gland. *Breast Cancer Res* **14**, 203, doi:10.1186/bcr3065 (2012).

9 Iravani, O., Bay, B. H. & Yip, G. W. Silencing HS6ST3 inhibits growth and progression of breast cancer cells through suppressing IGF1R and inducing XAF1. *Exp Cell Res* **350**, 380-389, doi:10.1016/j.yexcr.2016.12.019 (2017).

10 Yamada, S., Pokutta, S., Drees, F., Weis, W. I. & Nelson, W. J. Deconstructing the cadherin-catenin-actin complex. *Cell* **123**, 889-901, doi:10.1016/j.cell.2005.09.020 (2005).

11 Khoshgoo, N. *et al.* MicroRNA-200b regulates distal airway development by maintaining epithelial integrity. *Sci Rep* **7**, 6382, doi:10.1038/s41598-017-05412-y (2017).

12 Um, J. W. *et al.* LRRTM3 Regulates Excitatory Synapse Development through Alternative Splicing and Neurexin Binding. *Cell Rep* **14**, 808-822, doi:10.1016/j.celrep.2015.12.081 (2016).

13 Mota, C. *et al.* Human aldehyde oxidase (hAOX1): structure determination of the Moco-free form of the natural variant G1269R and biophysical studies of single nucleotide polymorphisms. *FEBS Open Bio* **9**, 925-934, doi:10.1002/2211-5463.12617 (2019).

14 Stanzione, M. *et al.* Meiotic DNA break formation requires the unsynapsed chromosome axis-binding protein IHO1 (CCDC36) in mice. *Nat Cell Biol* **18**, 1208-1220, doi:10.1038/ncb3417 (2016).

15 Haining, E. J. *et al.* The TspanC8 subgroup of tetraspanins interacts with A disintegrin and metalloprotease 10 (ADAM10) and regulates its maturation and cell surface expression. *J Biol Chem* **287**, 39753-39765, doi:10.1074/jbc.M112.416503 (2012).

16 Song, J. *et al.* Differential roles of human PUS10 in miRNA processing and tRNA pseudouridylation. *Nat Chem Biol* **16**, 160-169, doi:10.1038/s41589-019-0420-5 (2020).

17 Lee, M. Y. *et al.* Peroxisomal protein PEX13 functions in selective autophagy. *EMBO Rep* **18**, 48-60, doi:10.15252/embr.201642443 (2017).
